# Polypeptide-Based Molecular Platform and Its Docetaxel/Sulfo-Cy5-Containing Conjugate for Targeted Delivery to Prostate Specific Membrane Antigen

**DOI:** 10.3390/molecules25245784

**Published:** 2020-12-08

**Authors:** Stanislav A. Petrov, Aleksei E. Machulkin, Anastasia A. Uspenskaya, Nikolay Y. Zyk, Ekaterina A. Nimenko, Anastasia S. Garanina, Rostislav A. Petrov, Vladimir I. Polshakov, Yuri K. Grishin, Vitaly A. Roznyatovsky, Nikolay V. Zyk, Alexander G. Majouga, Elena K. Beloglazkina

**Affiliations:** 1 Department of Chemistry, Lomonosov Moscow State University, Leninskie Gory, 1-3, 119991 Moscow, Russia; stanislavpetrovsh1994@gmail.com (S.A.P.); alekseymachulkin@rambler.ru (A.E.M.); uspenskaya.n@gmail.com (A.A.U.); zyknikola@gmail.com (N.Y.Z.); nimenkoea@mail.ru (E.A.N.); anastasiacit@gmail.com (A.S.G.); petrovrostaleks@gmail.com (R.A.P.); grishin@nmr.chem.msu.ru (Y.K.G.); Vit.Rozn@nmr.chem.msu.su (V.A.R.); zyk@org.chem.msu.ru (N.V.Z.); alexander.majouga@gmail.com (A.G.M.); 2 Laboratory of Biomedical Nanomaterials, National University of Science and Technology MISiS, Leninskiy pr., 4, 119049 Moscow, Russia; 3 Faculty of Fundamental Medicine, Lomonosov Moscow State University, Lomonosovsky Ave., 27-1, 119991 Moscow, Russia; vpolsha@mail.ru; 4 Mendeleev University of Chemical Technology of Russia, Miusskaya sq. 9, 125947 Moscow, Russia

**Keywords:** theranostic agent, peptide synthesis, prostate cancer, anticancer drugs, PSMA conjugate, docetaxel, Sulfo-Cy5

## Abstract

A strategy for stereoselective synthesis of molecular platform for targeted delivery of bimodal therapeutic or theranostic agents to the prostate-specific membrane antigen (PSMA) receptor was developed. The proposed platform contains a urea-based, PSMA-targeting Glu-Urea-Lys (EuK) fragment as a vector moiety and tripeptide linker with terminal amide and azide groups for subsequent addition of two different therapeutic and diagnostic agents. The optimal method for this molecular platform synthesis includes (a) solid-phase assembly of the polypeptide linker, (b) coupling of this linker with the vector fragment, (c) attachment of 3-aminopropylazide, and (d) amide and carboxylic groups deprotection. A bimodal theranostic conjugate of the proposed platform with a cytostatic drug (docetaxel) and a fluorescent label (Sulfo-Cy5) was synthesized to demonstrate its possible sequential conjugation with different functional molecules.

## 1. Introduction

Prostate cancer (PCa) is one of the most commonly diagnosed men’s cancers and remains one of the leading causes of cancer death. In 2018, approximately 1,276,106 new cases and 358,989 suspected deaths were diagnosed worldwide [1,2].

Depending on the stage of the cancer and its severity, various imaging techniques, such as computed tomography (CT), transrectal ultrasound, and relatively recent methods such as magnetic resonance imaging (MRI), single-photon emission computed tomography (SPECT), and positron emission tomography (PET) are used to assess prostate cancer [3]. However, the specificity of existing imaging methods in evaluating metastases is limited [4,5]. The selected method for the treatment of PC usually depends on the stage of the disease. For example, for localized PC, the options range from radical prostatectomy to radiation therapy. Metastatic PC is preferably treated with androgen deprivation therapy (ADT). When tumors develop resistance to androgens, the options are reduced to alternative hormone therapy or chemotherapy. Preferred therapeutic agents are taxanes, such as docetaxel [6,7]. However, so far, no active treatment for PC showed superiority in survival rates. Treatment options differ only concerning their side effects.

One of the promising methods in PCa therapy and diagnostics is targeted delivery of medicinal and diagnostic drugs to cancer cells, as well as delivery of theranostics. Theranostic conjugates are drugs for the simultaneous solving of therapeutic and diagnostic problems. These compounds are composed of several functional units, one of which is an early diagnostic tool and the other is a therapeutic agent. This allows diagnosis and treatment of the disease simultaneously [8,9,10]. The advantage of this approach is the decrease in side effects and injection dose in order to improve diagnosis and treatment of the disease [11]. In addition, theranostic agents can minimize the inevitable differences in biodistribution and selectivity that exist between diagnostic and therapeutic materials for a particular disease [12]. This is particularly important in the case of cancer pathologies that are highly heterogeneous [13].

Due to the high expression of prostate-specific membrane antigen on the cell membrane of prostate cancer cells, this protein is an attractive molecular target for PCa theranostics [14]. Urea-based EuK inhibitor is currently the reference in the development of targeted delivery systems to a prostatic specific membrane antigen due to its stability, high affinity, and good bioavailability [15].

The study of the prostate-specific membrane antigen (PSMA) crystal structure showed that access to the binding site of this enzyme is provided by a ligand through a 20-Å-long, narrow tunnel with two hydrophobic pockets and an arginine cluster [16,17]. Therefore, to construct a sterically unhindered conjugate with optimal complementarity to the contours and chemical composition of the tunnel, the ligand must be associated with diagnostic and therapeutic fragments via a linker of a certain length and chemical composition. According to previous studies, relatively short lipophilic linkers are preferable, as well as the presence of aryl moieties in the linker, which significantly increase affinity. However, too much lipophilicity may adversely affect the selectivity of accumulation. In general, careful selection of the linker fragment allows optimization of the efficiency of PSMA inhibition, cellular internalization, accumulation in nontarget tissues (kidneys, liver, spleen, etc.), as well as the quality of visualization in vivo [18]. Thus, the chemical structure of the linker fragment has a significant influence on the affinity to PSMA, as well as on the pharmacokinetic properties of PSMA-oriented theranostic conjugates.

**Figure 1:**
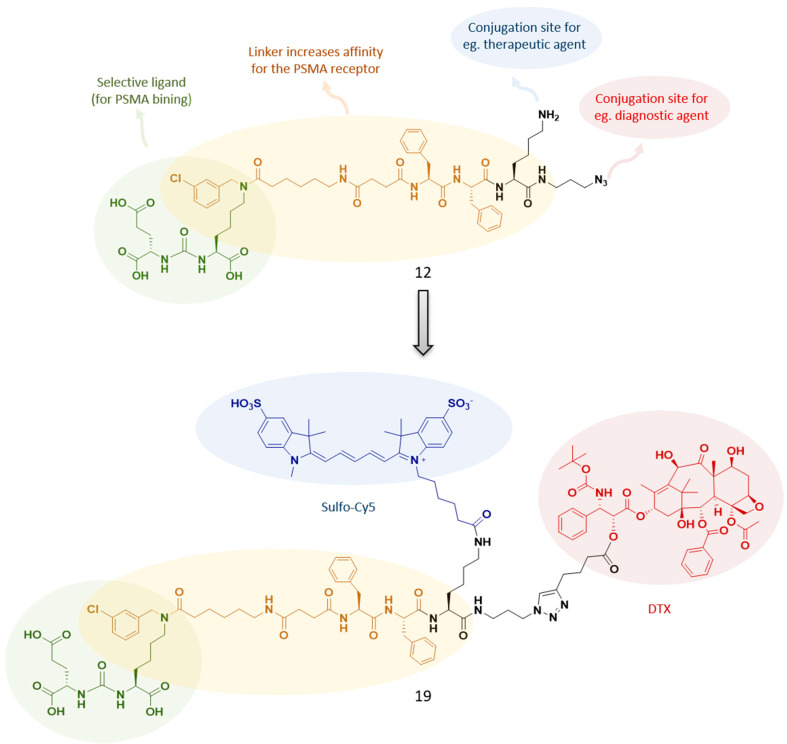
EuK-based ligand 12 with terminal amino and azido groups, synthesized in this work, and bimodal conjugate 19 on its base.

In this work, the development of synthetic approaches to create a molecular platform based on the EuK ligand for targeted delivery of bimodal therapeutic or theranostic agents specific to the PSMA receptor was carried out. The structures of synthesized compounds are shown in Figure 1. The proposed molecular platform for PSMA delivery consists of two parts: (1) a vector fragment providing conjugate-directed delivery to the prostate cancer cells and (2) a polypeptide linker providing a possibility of subsequent conjugation with therapeutic and diagnostic (or two therapeutic) agents and increasing affinity to the PSMA receptor. As part of the work, a comparison of liquid- and solid-phase techniques for synthesis of the delivery molecule was made.

To demonstrate the possibility of the synthesized bifunctional probe application for the stepwise attachment of diagnostic and therapeutic agents, a double conjugate with docetaxel (DTX) and a fluorescent dye Sulfo-Cyanine5 (Sulfo-Cy5) (Figure 1) was synthesized. This obtained compound was tested for cytotoxic activity and cell staining.

## 2. Results and Discussion

Previously, we described therapeutic conjugates of doxorubicin [19] and paclitaxel [20] with PSMA ligands of structurally related types, and it was shown that, for maximum affinity to the receptor, conjugate polypeptide fragments should contain aromatic substituents of different nature in the ζ-NH_2_ position of Lys-amino acid of the PSMA ligand and the dipeptide fragment Phe(L)-Phe(L) in the linker structure [18,19]. In this article, two functional groups of different nature were introduced into the linker fragment for further stage-by-stage conjugation, with diagnostic and therapeutic moieties at orthogonal conditions to obtain the bimodal theranostic agents. These groups were NH_2_, which allows attachment of the additional structural fragments using peptide synthesis reactions, and N_3_, which can be entered into azide–alkyne cycloaddition (Figure 1).

**Scheme 1:**
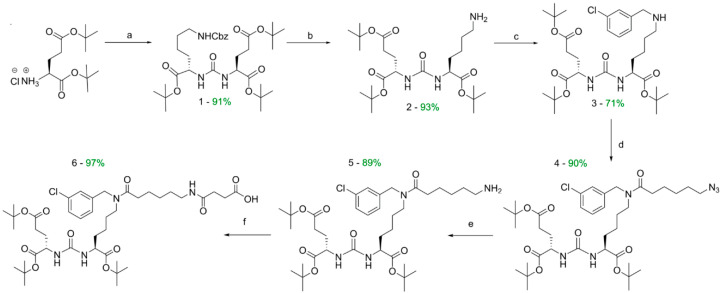
Synthesis of prostate-specific membrane antigen (PSMA)-vector fragment. Reagents and conditions: (**a**) (1) Thriphosgene, DCM (dichloromethane), −78 °C; (2) H-Lys(Cbz)-O-tBu·HCl, Et_3_N, 20 °C; (**b**) H_2_, Pd/C (10%), MeOH; (**c**) (1) 3-Cl--C_6_H_4_-CHO, DCM (2) NaBH_4_; (**d**) PyBOP (benzotriazol-1-yl-oxytripyrrolidinophosphonium hexafluorophosphate), DIPEA (N,N-diisopropylethylamine), DMF, N_3_(CH_2_)_5_COOH; (**e**) THF/H_2_O, Ph_3_P, 50 °C; (**f**) (1) succinic anhydride, DCM, DIPEA; (2) MeOH; (3) HCl (0.1M). All amino acids have L-configuration.

**Scheme 2:**
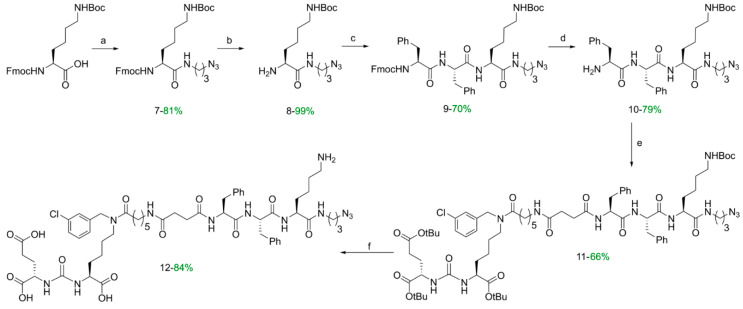
Synthesis of the vector fragment with the peptide linker by the liquid-phase technique. Reagents and conditions: (**a**) (1) HBTU (hexafluorophosphate benzotriazole tetramethyl uranium), HOBt (1-hydroxybenzotriazole), DIPEA, DMF; (2) N_3_(CH_2_)3NH_2_; (**b**) Et_2_NH, DMF; (**c**) HBTU, HOBt, DIPEA, FmocPhePhe-OH, DMF; (**d**) Et_2_NH, DMF; (**e**) (1) **6**, HBTU, HOBt, DIPEA, DMF; (2) **10**; (**f**) DCM/TFA (trifluoroacetic acid). All amino acids have L-configuration.

**Scheme 3:**
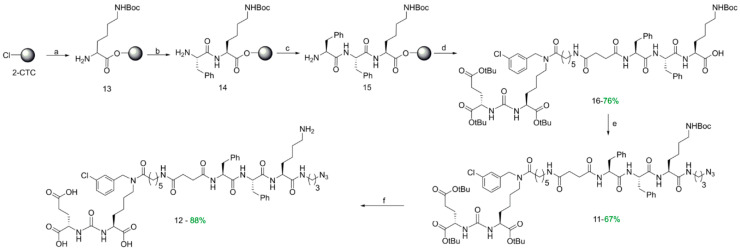
Synthesis of the vector fragment with the peptide linker by SPPS. Reagents and conditions: (**a**) (1)FmocLys(L)(NHBoc), DIPEA, DMF; (2) 4-methylpiperidine/DMF; (**b**) (1) FmocPhe-OH(L), HBTU, HOBt, DIPEA; (2) 4-methylpiperidine/DMF; (**c**) (1) FmocPhe-OH(L), HBTU, HOBt, DIPEA; (2) 4-methylpiperidine/DMF; (**d**) (1) 6, HBTU, HOBt, DIPEA, DMF; (2) DCM/TFA (99.25%/0.75%); (**e**) (1) HBTU, HOBt, DIPEA, DMF; N_3_(CH_2_)_3_NH_2_; (**f**) DCM/TFA/TIPS/H_2_O. All amino acids have L-configuration.

**Scheme 4:**
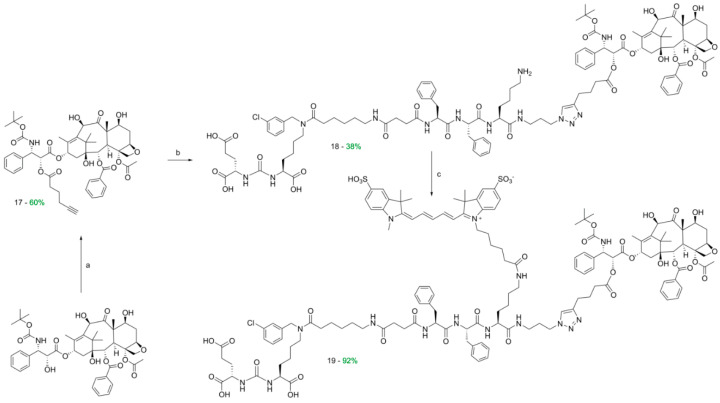
Synthesis of the docetaxel/Cy5-containing conjugate. Reagents and conditions: (**a**) Hex-5-ynoic acid, DMAP (4-Dimethylaminopyridine), DCM; (2) DIC (N,N′-Diisopropylcarbodiimide); (**b**) (1)**12**,**17**, CuSO_4_*5H_2_O, sodium ascorbate, DMF, H_2_O; (2) EDTA (Ethylenediaminetetraacetic acid); (**c**) (1) DIPEA, DMF; (2) Sulfo-Cy5-NHS ester.

To obtain the target PSMA ligand with peptide fragments, we developed the synthetic scheme, including the following stages: (1) synthesis of EuK vector **6** with modified urea fragment (Scheme 1), (2) synthesis of the tripeptide linker using liquid-phase techniques (Scheme 2), (3) alternative synthesis of the tripeptide linker using solid-phase peptide synthesis (SPPS) techniques (Scheme 3), (4) coupling of the vector fragment with the linker with the formation of compound **12** (Scheme 2 and Scheme 3), (5) modification of docetaxel with hex-5-ynoic acid giving intermediate **17** (Scheme 4), and (6) click reaction between the compounds **17** and **12** and the subsequent conjugation of the resulting compound with a fluorescent label (Scheme 4).

### 2.1. The Assembly of the Peptide Sequence

The initial stages of the synthesis of the vector fragment **6** (Scheme 1) were realized by previously described methods [21]. Compound 6 was prepared by coupling of succinic anhydrides with compound **5** (Scheme 1); the resulting products contained a free carboxylic group suitable for further addition of the peptide fragment.

Tripeptide (Phe(L)-Phe(L)-Lys(L)-(CH_2_)_3_-N_3_) was synthesized from L-phenylalanine (F) and L-lysine (K) to obtain highly specific PSMA vectors. Phe(L)–Phe(L) dipeptide fragments in the linker improve the binding to the receptor [18,19]; the dipeptide nature of linkers further improves biodegradability and reduces the unsystematic toxicity of PSMA vectors [22,23]. The coupling of additional lysine amino acid with an azide-containing fragment to the Phe(L)–Phe(L) linker provides the possibility of further modification with therapeutic and diagnostic drugs in orthogonal conditions.

#### 2.1.1. Synthesis of Tripeptide Sequence by Liquid-Phase Technique

The assembly of the peptide sequence was performed in the following manner (Scheme 2): Nα-Fmoc-Nε-Boc-l-lysine was introduced into the reaction with 3-aminopropylazide to obtain compound **7**, from which Fmoc was subsequently removed; as a result, the product containing a free amino group **8** was isolated. At the next step, a peptide synthesis between compounds **8** and Fmoc-PhePhe-OH was performed to synthesize the compound **9**. The removal of Fmoc protection allowed the desired compound **10** to be obtained as an individual stereoisomer (see Supplementary Information, Figure S4).

#### 2.1.2. Synthesis of Tripeptide Sequence by SPPS Technique

The assembly of the peptide sequence was also realized using solid phase peptide synthesis (SPPS) on 2-chlorotrityl chloride resin (2-CTC). This reaction sequence is presented as a classical peptide synthesis scheme: (1) immobilization of N-substituted amino acid on a solid-phase polymer substrate, (2) removal of the protective group, (3) modification of the NH_2_-group of the amino acid (stages 2 and 3 were repeated to get the desired peptide sequence), and (4) removal of obtained peptide from the polymer substrate [24].

Subsequently, the operations were performed with the necessary amino acids to obtain compound **15** (Scheme 3).

The 2-CTC resin allows application of the Fmoc SPPS concept and minimizes the adverse reactions. Furthermore, it keeps labile acid functional groups intact, since the amino acid sequence is removed from the polymer substrate under mild conditions (in our case DCM/TFA—99.25%/0.75%, *V*/*V*; this system does not affect labile acid actions of the NHBoc and COOtBu groups) [25].

### 2.2. Synthesis of DCL-Modified Tripeptide 12

For the coupling of the vector fragments with peptide sequences by liquid-phase technique vector compound **6** was dissolved in DMF and preactivated using the HOBt/HBTU/DIPEA system for 2 h (Scheme 2). Then, compound **10** was added and the mixture was stirred for 24 h. The reaction product **11** was isolated by column chromatography and further converted to compound **12** (see Section 2.4). All substances were obtained as individual stereoisomers (see Supplementary Information, Figures S7 and S8).

During the SPPS sequence (Scheme 3), vector fragment **6** was attached to tripeptide **15**, mounted on 2-CTC. After that, the modified tripeptide was removed from the polymer carrier by treatment with DCM/TFA. As a result, compounds **16** were isolated as individual stereoisomers according to the ^1^H NMR,^13^C NMR LCMS, HRMS data (see Supplementary Information, Figures S5 and S6).

Further, 3-aminopropylazide was attached to the free carboxyl group of compounds **16**. Based on published data, these reactions may be carried out by one of three possible procedures [26]:

1. Addition of a coupling reagent (carbodiimide, EEDQ (N-Ethoxycarbonyl-2-ethoxy-1,2-dihydroquinoline), phosphonium and carbenium salts, trisubstituted phosphates, etc.) and a tertiary amine, if necessary, to a mixture of the acid and the amine nucleophile to be combined;
2. Addition of the amine nucleophile to a solution of the coupling reagent and the acid only after they were reacted and an activated compound was generated;
3. Addition of the amine nucleophile to one of the activated forms of the acid (activated ester, acyl azide, anhydrides, etc.) to which it was to be combined.

Considering method 1, it is necessary to note that the activated agent (HBTU) is capable of reacting with N-terminal amino component, leading to a guanidine derivative; this side process may compete for peptide chain elongation. To avoid this side reaction, the preliminary activation of the carboxylic acid component is recommended [27]. We performed method 1 (addition of a coupling reagent and tertiary amine to a mixture of the acid and the amine). Applying this technique to the reaction of compound **16** with 3-aminopropylazide, we obtained the individual stereoisomer of desirable substance **11,** as confirmed by NMR spectroscopy (see Supplementary Information, Figure S8).

Taking into account the racemization taking place during the discussed reactions, we concluded that method 2 (adding amine to the activating agent solution, tertiary amine, and acid) was not optimal for stereoselective syntheses of individual stereoisomer of target peptide due to possible intermediate formation of achiral oxazolone intermediate [26]. However, method 2 could be successfully used to obtain compound **11** by the liquid-phase technique (Scheme 2). This is explained by the fact that in the case of the liquid-phase technique, the carboxylic group involved in the formation of a peptide bond is in a vector fragment and does not have a stereocenter in the α-position. Therefore, the possible formation of oxazolone during the reaction does not lead to racemization. NMR spectra of compound **11** obtained by the liquid-phase technique are given in the Supplementary Information (Figure S7).

When using method 3, it should be noted that there is no general method for activated amino acid creation. Also, it is necessary to activate the acid with this method, and then isolate the activated form, which adds an extra stage of synthesis and may lead to undesirable reactions with inappropriate functional groups [26]. For this reason, we did not test method 3 to obtain compound **12**.

The next stage of the synthesis was the removal of the protective tert-butyl groups from carboxyl fragments and the Boc group from ε-NH_2_ of terminal lysine moiety (Scheme 2 and Scheme 3). The deprotection was performed by two methods, i.e., by treating of compound **11** with TFA/DCM or DCM/TFA/TIPS/H_2_O mixture. As a result, target compound **12** was obtained, and its structure was confirmed by HRMSm as well as ^1^H/^13^C NMR (see Supplementary Information, Figures S9–S12).

The data obtained for different methods of vector peptide **12** synthesis are summarized in Table 1. The total yields of the target compounds based on the starting amino acid for linker formation and the starting Boc-Fmoc-protected lysine (Scheme 1) were evaluated. The laboriousness of the syntheses was compared, taking into account the total number of synthetic stages and the number of stages with chromatographic isolation of the target product.

In summary, the liquid-phase technique (Scheme 2) using method 2 (addition of the amine nucleophile to a solution of the coupling reagent and the acid only after they were reacted and generated an activated compound) to create a peptide bond between compound **10** and a vector fragment of ligand **6** was characterized by a maximum yield based on compound **11,** but the minimum yield counting of the initial amino acid. The total number of stages using laborious chromatographic isolation was also large.

SPPS technique (Scheme 3) using method 1 (addition of a coupling reagent and tertiary amine to a mixture of the acid and the amine) to create a peptide bond between **16** and 3-aminopropylazide, showed the best yield on the initial amino acid and good yield on compound **6**, which seemed to be optimal. Also, this technique showed further advantages over the liquid-phase technique, namely, a less time-consuming process of target platforms obtaining, product isolation simplicity, and the absence of additional purification stages, both for intermediate compounds and the target substance.

However, it should be noted that the liquid-phase technique allows the obtainment of large amounts of target compounds, although it is more laborious and time-consuming than the SPPS approaches. At the same time, obtainment by the SPPS technique may be convenient for the rapid preparation of the libraries of similar compounds, although the reactions proceed with lower yields and require a large excess of amino acids.

### 2.3. Synthesis of the Bimodal Conjugate 19

At the next stage of the work, to demonstrate the possibility of compound **12** use as a molecular platform for bimodal agent preparation, we synthesized its double conjugate with the anticancer drug docetaxel and a fluorescent dye Sulfo-Cy5. Docetaxel is a taxane-derivative diterpenoid and is one of the most widely used anticancer agents in clinical practice today [28]. Analysis of literature data on the effect of modifications of various structural fragments of docetaxel on its activity suggested that the most appropriate strategy for introducing a linker is to form an ester bond with one of the secondary hydroxyl groups [29]. In cells, the ester bond is known to hydrolyze with the extrication of free drug. We carried out the reaction of docetaxel with hex-5-ynoic acid, and the obtained adduct **17** was also introduced into the azide–alkyne cycloaddition with peptide **12**. The standard procedure for ester formation in the presence of diisopropylcarbodiimide (DIC) and a catalytic amount of 4-(dimethylamino)pyridine (DMAP) gave compound **17** with reasonable yield (Scheme 4). 2D NMR spectroscopy (HSQC ^1^H-^13^C, HMBC ^1^H-^13^C) made it possible to make complete signal correlation in the spectra of compound **17** (Supplementary Information, Figures S13–S16, Tables S1 and S2).

To obtain conjugate **18** from azide **12** and alkyne **17**, we chose the click-reaction of 1,3-dipolar cycloaddition catalyzed by copper(I). This reaction is widely used in synthesizing biologically active organic compounds, in particular, agents against tuberculosis and peptide–carbohydrate conjugates [30]. The complete correlation of signals in NMR spectra of compound **18** was made using 2D NMR spectroscopy (HSQC ^1^H-^13^C, HMBC ^1^H-^13^C; see Supplementary Information, Figures S17–S20, Tables S3 and S4).

At the next step, the NHS-activated ester of the fluorescent label Sulfo-Cy5 was attached to the free NH_2_ group of compound **18**. Near-infrared fluorescence (NIRF) imaging agents, like Sulfo-Cy5, have high extinction coefficients, large Stokes’ shifts, and are able to generate strong fluorescence emission offering the possibility of in vivo cancer diagnosis. Their considerable advantages for in vivo imaging include stronger ligand labeling, signal strength, and tissue absorbance, a wider range of imaging materials for coupling, and less background fluorescence. The far-red cyanine dye Sulfo-Cy5 (λ_ex_ 640 nm, λ_em_ 656 nm), with high detection sensitivity (0.05 vs. 3.15 mM for Indocyanine green (ICG)), tissue penetration (9 vs. 6 mm for ICG), and brightness (quantum yield, 28% vs. 0.3% for ICG) [31], was chosen as the fluorescent label for conjugation with **18**.

As a result, the target bimodal conjugate **19** was obtained, and its structure was confirmed by HRMS, LCMS, and ^1^H-NMR data. Moreover, initial biological experiments on the synthesized conjugate interaction with human cells differing in the level of PSMA expression were carried out in order to preliminary estimate its possibility and potential for biomedical application.

### 2.4. Biological Evaluation

**Figure 2:**
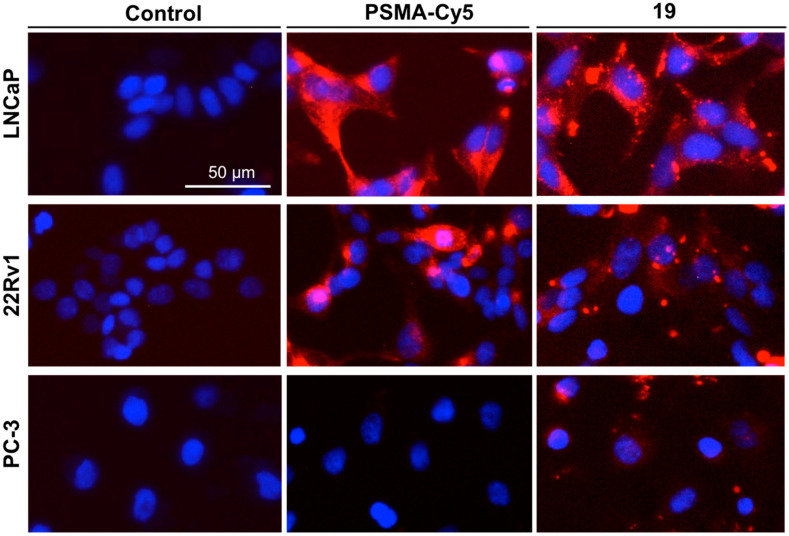
Interaction potency of the fluorescent conjugate **19** by LNCaP, 22Rv1, and PC-3 cells after 2 h of co-incubation. Cell nuclei are stained with blue 4′,6-diamidino-2-phenylindole (DAPI), a fluorescent stain that binds strongly to DNA. Fluorescent microscopy.

First, we investigated the selectivity of synthesized fluorescent conjugate **19** modified with PSMA vector toward three human prostate cancer cell lines, which differ in the level of PSMA expression—LNCaP (PSMA++), 22Rv1 (PSMA+), and PC-3 (PSMA-) [32]—using fluorescent microscopy. The results are presented in Figure 2.

We observed a homogeneous diffuse staining of all cells in the LNCaP line and a part of the cell population in 22Rv1 culture after 2 h incubation with PSMA-Cy5. It must be noted that the most intensive fluorescence signal from PSMA-Cy5 conjugate was observed in the perinuclear area. This fact indicated the intracellular localization of the PSMA-Cy5 conjugate. No fluorescence signal from PSMA-Cy5 was detected in cells of PSMA-negative PC-3 line. For compound **19**, all LNCaP cells were also positively stained. However, the nature of the staining was different—we revealed the fluorescent signal of conjugate **19** to be mainly point-concentrated, presumably, in cell vesicles. At the same time, less pronounced diffuse staining of the entire cells cytoplasm was found. A similar result was obtained for the 22Rv1 cell line. However, fluorescent signal from conjugate **19** was detected not in all cells of population. Moreover, the presence of point-concentrated localization of compound **19** in these cells was significantly less than in the LNCaP line. Single accumulations of conjugate **19** were identified predominantly in the lamellae of PC-3 cells. Thus, the obtained data demonstrated that the effectiveness of the conjugate **19** selective interaction with PSMA++ LNCaP cells was higher than with PSMA+ 22Rv1 cells. Interaction of compound **19** with PSMA- PC-3 cells was significantly lower than with both investigated PSMA-positive cell lines. Some accumulations of conjugate **19** revealed in PC-3 cells could be due to its nonspecific interaction with the cells and penetration by diffusion presumed for Docetaxel. The mechanism of this taxane penetration inside the cells was well studied only for hepatocytes, while further investigations are required for other cell types [33].

**Figure 3:**
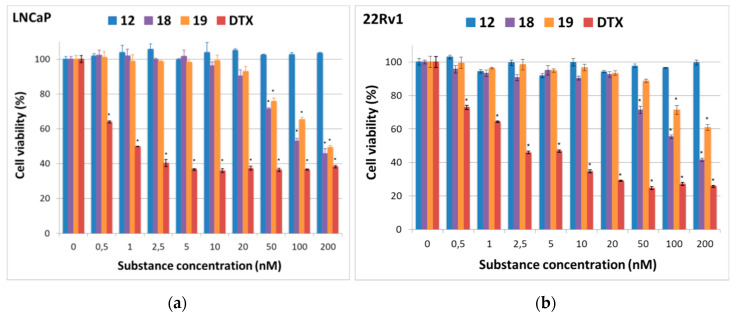
Cytotoxicity of compounds **12**, **18**, **19,** and docetaxel (DTX) against LNCaP (**a**) and 22Rv1 (**b**) tumor cells. Results are shown as means ± SD (*t*-test, * *p* < 0.05). MTS-assay. Experiments were performed in triplicate.

Further, bimodal conjugate **19**, as well as its synthetical precursors (conjugate **18**, containing docetaxel, but not containing a fluorescent label, peptide vector **12** without imaging or therapeutic agents, and free docetaxel as a comparison substance) were evaluated for in vitro cytotoxicity against two PSMA-positive cell lines—LNCaP and 22Rv1 (Figure 3) [32]. The cargo Docetaxel and conjugate **18** were used as a positive control, whereas compound **12** was used as a negative control. As a result, conjugates **18** and **19** showed good activity against both cell lines with a slightly more pronounced effect on LNCaP cells, where LNCaP IC_50_ = 100 nM and 200 nM, respectively, as well as 22Rv1 IC_50_ = 130 nM and >200 nM. Docetaxel by itself caused significant cell death in both cultures, where LNCaP IC_50_ = 1 nM and 22Rv1 IC_50_ = 2.1 nM. These data were consistent with the selectivity of the resulting conjugates in relation to cell lines expressing PSMA. The lower toxicity of conjugates **18** and **19** in comparison with free Docetaxel, could apparently be explained by the slow release of the active drug from the conjugate, consistent with previously obtained results [19]. Vector peptide **12**, as expected, was not toxic for either of the cell lines.

Based on these data, we can conclude that the designed vector is a perspective conjugate, which demonstrated selectivity and toxicity against PSMA-positive cells and should be further investigated in more detail for targeted drug delivery, at least in PSMA-overexpressed LNCaP cells.

## 3. Materials and Methods

All used solvents were purified according to procedures described in [34]. All starting compounds were commercially available reagents. The initial stages of the synthesis of the vector fragment **1**–**5** (Scheme 1) were made by methods previously developed by our scientific group [21]. Spectral data of the compounds **7** and **8** (Scheme 2) were described in [35]. ^1^H NMR was measured using a Bruker Avance spectrometer operating at 400 MHz for ^1^H using CDCl_3_ and DMSO-d_6_ as solvents. Chemical shifts were reported in δ units to 0.01 ppm precision with coupling constants reported to 0.1 Hz precision using residual solvent as an internal reference.^13^C NMR was measured using a Bruker Avance spectrometer operating at 100 MHz using DMSO-d_6_ as solvents. Chemical shifts were reported in δ units to 0.1 ppm precision using residual solvent as an internal reference. 2D NMR was measured using an Agilent 600 spectrometer operating at 600 MHz for ^1^H and 100 MHz for (^13^C) using DMSO-d_6_ as the solvent. As 2D NMR methods were used, such as heteronuclear single quantum coherence spectroscopy ^1^H-^13^C (gHSQC) and heteronuclear multiple bond correlation ^1^H-^13^C (gHMBC). NMR spectra were processed and analyzed using Mnova software (Mestrelab Research, Spain). High-resolution mass spectra were recorded on the Orbitrap Elite high-resolution mass spectrometer. Solutions of samples in acetonitrile with 1% formic acid were introduced into the ionization source by electrospray. For the HPLC analysis system with Shimadzu Prominence, an LC-20 column and a convection fraction collector connected with a single quadrupole mass spectrometer Shimadzu LCMS-2020 with dual ionization source DUIS-ESI-APCI were used. The analytical and preparative column was Phenomenex Luna 3u C18 100A. Preparative chromatographic separation of substances was carried out using the INTERCHIM puriFlash 430 chromatograph.

**Figure 4:**
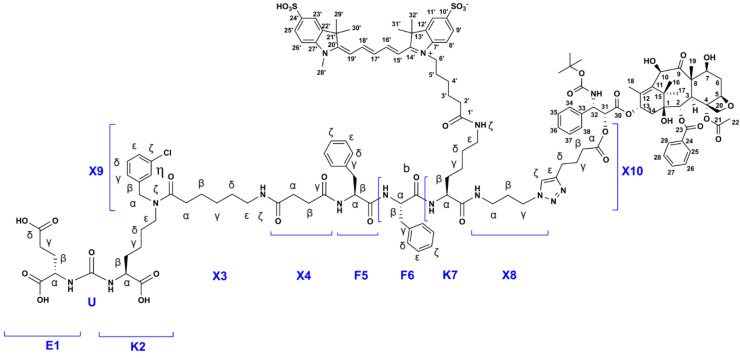
The notation of structural fragments of synthesized compounds (for conjugate **19**). E1 = glutamic amino acid residue, K2 = lysine amino acid residue etc.; a, b: diastereotopic protons; m, n: notation for two forms of rotational isomers, m/n = 3/2; Hα = Ha, Hβ = Hb etc.

For better interpretation of the NMR spectra of target compound **19**, the notation of structural fragments is shown in Figure 4.

Cell Lines: LNCaP, 22Rv1, and PC-3 human prostate cancer cells were purchased from the American Type Culture Collection (ATCC, Manassas, VA, USA).

Cell Cultivation: Cells were maintained in RPMI-1640 medium (gibco), supplemented with 10% Fetal Bovine Serum (Sigma), 2 mM L-glutamine, and RPMI vitamin solution (Sigma). Cells were cultured at 37 °C in a humidified incubator (Sanyo) supplied with 5% CO_2_. Cells were seeded on glass coverslips or in 96-well plates (Corning) at concentrations of 120,000 cells per mL for LNCaP, 200,000 cells per mL for 22Rv1, and 90,000 cells per mL for PC-3 in experiments. The counting of cells was carried out using the automatic cell counter EVE. 

Cell Incubation with Conjugates: A day after seeding the cells on glass coverslips, PSMA-Cy5 or fluorescently labeled compound **19** were added in culture medium at a concentration of 30 nM for 2 h. Later, cells were washed with PBS (pH 7.2–7.4) and fixed with 4% formaldehyde (Sigma) (on PBS) for 15 min. Cell nuclei were stained with DAPI (Sigma) for 10 min. Obtained preparations were imaged using an inverted fluorescence microscope EVOS (life technologies, objective PlanFluor 20×/0.45). Further processing of the photos was carried out by ImageJ software.

Cytotoxicity Assay: A day after cell seeding in 96-well plates, serial dilutions of conjugates and Docetaxel in culture medium were added to cells. Cells incubated in culture medium were used as control. DMSO diluted in the cell medium (20%) was used as a positive control. Cells were incubated for 72 h at 37 °C and 5% CO_2_. Later, the culture medium from each well was removed and 20 μL of MTS reagent (CellTiter 96 AQueous Non-Radioactive Cell Proliferation Assay, Promega) was added to each well with 100 μL of new culture medium. After 4 h of incubation at 37 °C in darkness, the absorbance of the obtained solution was measured at 490 nm wavelength using the Thermo Scientific Multiskan GO spectrometer. Cell viability was calculated as percent compared to cells incubated in culture medium. MTS assay revealed 100% cell death after incubation with 20% DMSO (data not shown). The absorbance of MTS reagent in culture medium without cells was taken as zero. Experiments were performed in triplicate.

**Compound 6**. To a solution of compound **5** (1 eq; 725 mg; 1.0 mmol) in 20 mL of DCM, DIPEA (1.4 eq; 244 μL; 1.4 mmol) and succinic anhydride (1.02 eq; 102 mg; 1.02 mmol) were added. The mixture was stirred for 12 h. After that, MeOH (2 eq.) was added and the resulting mixture was stirred for 1 h. Then, the solvent was removed under reduced pressure, and residue was dissolved in DCM and extracted with (1) 0.1 M HCl (2 × 30 mL) and (2) brine (2 × 30 mL). Then, the organic fraction was dried over Na_2_SO_4_, and concentrated under reduced pressure to obtain the final compound **6** as a yellow oil (801 mg, yield 97%).

^1^H-NMR (400 MHz, DMSO-*d6*, δ): 12.06 (br.s., 1H, X4C(O)OH), 7.81 (t, *J* = 5.2 Hz, *m*) & 7.77 (t, *J* = 5.2 Hz, *n*) (1H, X3NHk, *m* + *n*, *m*/*n* = 3/2), 7.40 (t, *J* = 7.7 Hz, X8He, *n*), 7.37–7.27 (m, X8Hd + X8He(*m*)), 7.26–7.21 (m, 1H, X8Ht, *m* + *n*), 7.19–7.10 (m, 1H, X8Hg, *m* + *n*), 6.34–6.20 (m, 2H, K2NH + E1NH, *m* + *n*), 4.56 (s, *n*) & 4.48 (s, *m*) (2H, X8Ha, *m* + *n*, *m*/*n* = 3/2), 4.07–4.00 (m, 1H, E1Ha, *m* + *n*), 4.00–3.90 (m, 1H, K2Ha, *m* + *n*), 3.22 (t, *J* = 7.3 Hz, *n*) & 3.19 (t, *J* = 7.3 Hz, *m*) (2H, K2He, *m* + *n, m*/*n* = 3/2), 3.01 (q, *J* = 6.4, 12.7 Hz, *m*) & 2.96 (q, *J* = 6.4, 12.7 Hz, *n*) (2H, X3He, *m* + *n*, *m*/*n* = 3/2), 2.44–2.38 (m, 2H, X4Hb, *m* + *n*), 2.36 (t, *J* = 7.4 Hz, X3Ha, *m*), 2.31–2.25 (m, 2H, X4Ha, *m* + *n*), 2.25–2.15 (m, E1Hg + X3Ha(*n*)), 1.91–1.80 (m, 1H, E1Hb(a)), 1.72–1.63 (m, 1H, E1Hb(b)), 1.63–1.56 (m, 1H, K2Hb(a)), 1.40–1.35 (m, 27H, tBu), 1.56–1.15 (m, 11H, K2Hb(b) + X3Hb + X3Hd+ K2Hd + K2Hg + X3Hg, *m* + *n*).

^13^C-NMR (100 MHz, DMSO-*d_6_*, δ): 173.93 (X4Cg), 172.26 (K2C(*n*)), 172.23 (K2C(*m*)), 172.22 (X3C(*n*)), 172.19 (X3C(*m*)), 171.95 (E1C), 171.47 (E1Cg), 170.76 (X4C(*m*)), 170.73 (X4C(*n*)), 157.18 (U(*m*)), 157.16 (U(*n*)), 141.20 (X9Cb(*m*)), 140.80 (X9Cb(*n*)), 133.45 (X9Ce(*n*)), 133.10 (X9Ce(*m*)), 130.63 (X9Cd(*n*)), 130.26 (X9Cd(*m*)), 127.24 (X9Ct(*m*)), 127.17 (X9Ck(*n*)), 126.88 (X9Ck(*m*)), 126.34 (X9Ct(*n*)), 126.08 (X9Cg(*m*)), 124.99 (X9Cg(*n*)), 80.59 (E1tBu), 80.42 (K2tBu(*m*)), 80.33 (K2tBu(*n*)), 79.77 (E1dtBu), 53.01 (K2Ca(*n*)), 52.88 (K2Ca(*m*)), 52.20 (E1Ca(*m*)), 52.18 (E1Ca(*n*)) 49.63 (X9Ca(*n*)), 47.11 (X9Ca(*m*)), 46.83 (K2Ce(*m*)), 45.20 (K2Ce(*n*)),38.49 (X3Ce(*m*)), 38.43 (X3Ce(*n*)), 32.34 (X3Ca(*n*)), 31.95 (X3Ca(*m*)), 31.83 (K2Cb), 30.93 (E1Cg), 30.06 (X4Ca), 29.25 (X4Cb), 29.13 (X3Cd(*m*)), 29.04 (X3Cd(*n*)), 27.75 (tBuE1), 27.69 (K2Cd(*m*)), 27.66 (tBuK2), 27.64 (tBuE1g + E1Cb), 26.72 (K2Cd(*n*)), 26.23 (X3Cg(*m*)), 26.15 (X3Cg(*n*)), 24.76 (X3Cb(*m*)), 24.63 (X3Cb(*n*)), 22.45 (K2Cg(*n*)), 22.27 (K2Cg(*m*)).

ESI-MS C_41_H_65_ClN_4_O_11_: *m*/*z* calcd. for [M + H^+^]^+^: 825.44, found: 825.45.

**Compound 7**. To a solution of **FmocLys(L)(NHBoc)-OH** (1 eq.; 1000 mg; 2.134 mmol) in DMF (20 mL), DIPEA (1.5 eq.; 556 μL; 3.2 mmol), HOBt (1.2 eq.; 344 mg; 2.56 mmol), and HBTU (1.2 eq.; 971 mg; 2.56 mmol), were added, then the resulting mixture was purged with Ar and stirred for 60 min. Then, NH_2_-(CH_2_)_3_-N_3_ (2 eq.; 38 mg; 0.38 mmol) was added and the mixture was stirred for 24 h under Ar atmosphere. At the next step, the solvent was removed under reduced pressure and re-evaporated with DCM twice. The residue was dissolved in DCM (50 mL) and extracted with 1) H_2_O (2 × 50 mL) and 2) brine (2 × 50 mL). Then, the organic fraction was dried over Na_2_SO_4_. After the solvent was removed under reduced pressure, the residue was purified by column chromatography (Puriflash 15μ 40g, eluent: Hex(100%)/EtOAc(0%) => Hex(0%)/EtOAc(100%) for 30 min. The eluent for TLC was EtOAc/Hex = 1:1. Compound **7** was obtained as a yellow oil (950 mg, 81% yield).

^1^H-NMR (400 MHz, CDCl_3_, δ): 7.76 (d, *J* = 7.5 Hz, 2H, Fmoc), 7.58 (d, *J* = 7.5 Hz, 2H, Fmoc), 7.40 (t, *J* = 7.4 Hz, 2H, Fmoc), 7.31 (t, *J* = 7.4 Hz, 2H, Fmoc), 6.43 (br.s., 1H, X8NH), 5.54 (br.d., 1H, K7NH), 4.63 (br.s., 1H, K7NHk), 4.40 (d, *J* = 6.8 Hz, 2H, Fmoc), 4.20 (t, *J* = 6.8 Hz, 1H, Fmoc), 4.15–4.01 (m, 1H, K7Ha), 3.40–3.25 (m, 4H, X8Hg + X8Ha), 3.18–3.01 (m, 2H, K7He), 1.92–1.81 (m, 1H, K7Hb(a)), 1.80–1.71 (m, 2H, X8Hb), 1.70–1.57 (m,1H, K7Hb(b)), 1.56–1.46 (m, 2H, K7Hd), 1.43 (s, 9H, tBu), 1.39–1.29 (m, 2H, K7Hg).

**Compound 8**. To a solution of **7** (1 eq.; 792 mg; 1.44 mmol) in DMF (10 mL), Et_2_NH (10 eq.; 1053 μL; 14.4 mmol) was added, then the resulting mixture was purged with Ar and stirred for 1 h. The control of the reaction was performed with TLC. The eluent for TLC was EtOAc/Hex = 1:1. At the next step, the solvent was removed under reduced pressure and re-evaporated with DCM twice. The residue was purified by column chromatography (Puriflash 15μ 25g, eluent: DCM(100%)/MeOH(0%) => DCM(85%)/MeOH(15%) for 30 min, after MeOH (100%) for 5 min. Compound **8** was obtained as a yellow oil (469 mg, 99% yield).

^1^H-NMR (400 MHz, CDCl_3_, δ): 4.59 (br.s., 1H, K7NHk), 3.40–3.25 (m, 5H, X8Hg + K7Ha + X8Ha), 3.19–3.02 (m, 2H, K7He), 1.91–1. 1.70 (m, 4H, K7Hb(a) + X8Hb + K7Hb(b)), 1.56–1.46 (m, 2H, K7Hd), 1.44 (s, 9H, tBu), 1.39–1.32 (m, 2H, K7Hg).

**Compound 9**. To a solution of **FmocFF** (1 eq.; 770 mg; 1.44 mmol) in DMF (20 mL), DIPEA (1.2 eq.; 301 μL; 1.73 mmol), HOBt (1.2 eq.; 233 mg; 1.73 mmol), HBTU (1.2 eq.; 655 mg; 1.73 mmol), and **8** (1 eq.; 469 mg; 1.43 mmol) were added, then the resulting mixture was purged with Ar and stirred for 16 h. The control of the reaction was performed with TLC. The eluent for TLC was DCM/MeOH = 19:1. At the next step, the solvent was removed under reduced pressure and re-evaporated with DCM twice. The residue was dissolved in DCM (50 mL), and extracted with 1) H_2_O (2 × 50 mL), 2) brine (2 × 50 mL). Then, the organic fraction was dried over Na_2_SO_4_. After the solvent was removed under reduced pressure, the residue was purified by column chromatography (Puriflash 15μ 40g, eluent: DCM(100%)/MeOH(0%) => DCM(90%)/MeOH(10%) for 30 min, after MeOH (100%) for 5 min. Compound **9** was obtained as a yellow oil (853 mg, 70% yield).

**Compound 10**. To a solution of **9** (1 eq.; 840 mg; 0.995 mmol) in DMF (7 mL), Et_2_NH (10 eq.; 1029 μL; 9.95 mmol) was added, then the resulting mixture was purged with Ar and stirred for 1 h. The control of the reaction was performed with TLC. The eluent for TLC was DCM/MeOH = 19:1. At the next step, the solvent was removed under reduced pressure and re-evaporated with DCM twice. The residue was purified by column chromatography (Puriflash 15μ 25g, eluent: DCM(100%)/MeOH(0%) => DCM(90%)/MeOH(10%) for 30 min, after MeOH (100%) for 5 min. Compound **10** was obtained as a yellow oil (490 mg, 79% yield).

^1^H-NMR (400 MHz, DMSO-*d_6_*, δ): 8.14 (d, *J* = 7.9 Hz, 1H, F6NH), 8.06 (d, *J* = 7.7 Hz, 1H, K7NH), 7.90 (t, *J* = 5.7 Hz, 1H, X8NH), 7.29–7.09 (m, 10H, Ph + Ph), 6.77 (t, *J* = 5.5 Hz, 1H, K7NHk), 4.64–4.54 (m, 1H, F6Ha), 4.21–4.09 (m, 1H, K7Ha), 3.40–3.29 (m, 4H, F5Ha + F6Hb(a) + X8Hg), 3.11 (q, *J* = 6.0, 6.5 Hz, 2H, X8Ha), 2.97 (dd, *J* = 13.8, 4.9 Hz, 1H, F6Hb(b)), 2.91–2.78 (m, 4H, F5Hb(ab) + K7He), 1.73 (br.s., 2H, F5NH_2_), 1.69–1.56 (m, 3H, X8Hb + K7Hb(a)), 1.56–1.44 (m, 1H, K7Hb(b)), 1.36 (s, 9H, tBu), 1.35–1.29 (m, 2H, K7Hd), 1.28–1.11 (m, 2H, K7Hg).

**Compound 15.** Activation of 2-CTC. The mixture of 2-CTC (1 eq.; 1 g; 1.2–1.4 mmol/g; 100–200 mesh) in DCM (10 mL) was stirred for 10 min, then the mixture was purged with Ar, then SOCl_2_ (3 eq.; 305 µL; 4.2 mmol) was added dropwise, and then DMF (16 µL; 5% *V*/*V* to SOCl_2_) was added and stirred at 40 °C for 4 h. After that, the resin was filtered and transferred to a polypropylene reactor and washed with DMF (3 × 10 mL, 1 min) and DCM (3 × 10 mL, 1 min).

The addition of FmocLys(L)(NHBoc)-OH. To the mixture of CTC-2 (1 equiv; 1 g; 1.2–1.4 mmol/g; 100–200 mesh) in DMF (10 mL), FmocLys(NHBoc)-OH (2 eq.; 1.312 g; 2.8 mmol) and DIPEA (10 eq.; 2.44 mL; 14 mmol) were added, and the mixture was stirred for 2 h. Then, the resin was filtered off and washed with MeOH (3 × 10 mL, 5 min), DCM (3 × 0 mL, 1 min), DMF (3 × 10 mL, 1 min), and DCM (3 × 10 mL, 1 min).

Deprotection of Fmoc. FmocK(NHBoc) on a 2-CTC resin (1 eq.) was washed with DMF (2 × 15 mL, 1 min), then 4-methylpiperidine in DMF (20%/80% *V*/*V*, 15 mL) was added and stirred for 15 min, then the resin was filtered off and washed with DMF (3 × 15 mL, 1 min), then 4-methylpiperidine in DMF (20%/80% *V*/*V*,15 mL) was added and stirred for 15 min. After the resin was filtered off, the resulting solution was washed with DMF (3 × 15 mL, 1 min) and DCM (3 × 15 mL, 1 min).

The addition of FmocPhe(L)-OH. To the mixture of NH_2_-K(NHBoc) on a CTC-2 resin (1 eq.) in DMF (15 mL), FmocPhe(L)-OH (2 eq.; 1.085 g; 2.8 mmol), HOBt (0.5 eq.; 95 mg; 0.7 mmol), HBTU (2 eq.; 1.062 g; 2.8 mmol), and DIPEA (3 eq.; 0.73 mL; 4.2 mmol) were added and stirred for 2 h. Then the resin was filtered off and washed with DMF (3 × 15 mL, 1 min) and DCM (3 × 15 mL, 1 min).

Deprotection of Fmoc. FmocFK(NHBoc) on a CTC-2 resin (1 eq.) was washed with DMF (2 × 15 mL, 1 min), then 4-methylpiperidineine in DMF (20%/80% *V*/*V*, 15 mL) was added and stirred for 15 min, then the resin was filtered off and washed with DMF (3 × 15 mL, 1 min), then 4-methylpiperidine in DMF (20%/80% *V*/*V*, 15 mL) was added and stirred for 15 min. After the resin was filtered off, DMF (3 × 15 mL, 1 min) and DCM (3 × 15 mL, 1 min) wash was carried out.

Addition of FmocPhe(L)-OH. To the mixture of NH_2_-FK(NHBoc) on a CTC-2 resin (1 eq.) in DMF (15 mL), FmocPhe(L)-OH (2 eq.; 1.085 g; 2.8 mmol), HOBt (0.5 eq.; 95 mg; 0.7 mmol), HBTU (2 eq.; 1.062 g; 2.8 mmol), and DIPEA (3 eq.; 0.73 mL; 4.2 mmol) were added and stirred for 2 h. Then, the resin was filtered off and washed with DMF (3 × 15 mL, 1 min) and DCM (3 × 15 mL, 1 min).

Deprotection of Fmoc. FmocFFK(NHBoc) on a CTC-2 resin (1 eq.) was washed with DMF (2 × 15 mL, 1 min), then 4-methylpiperidineine in DMF (20%/80% *V*/*V*, 15 mL) was added and stirred for 15 min. Then, the resin was filtered off, washed with DMF (3 × 15 mL, 1 min), then 4-methylpiperidine in DMF (20%/80% *V*/*V*, 15 mL) was added and stirred for 15 min. After the resin was filtered off, DMF (3 × 15 mL, 1 min) and DCM (3 × 15 mL, 1 min) wash was carried out. Thus, the NH_2_-FFK(NHBoc) tripeptide was obtained on 2-CTC resin (1.95 g, ~1.4 mmol).

**Compound 16**. To the mixture of tripeptide **15** NH_2_-F5F6K7(NHBoc) on 2-CTC resin (1 eq.; 463 mg; 0,33 mmol) in DMF (5 mL) in a polypropylene reactor, compound **6** (1.2 eq.; 327 mg; 0,396 mmol), HOBt (0.5 eq.; 22 mg; 0.165 mmol), HBTU (2 eq.; 250 mg; 0.66 mmol), and DIPEA (3 eq.; 172 μL; 0.99 mmol) were added. The mixture was stirred for 2 h. Then, the solvent was removed by filtration on a porous reactor filter and the resin was washed with DMF (3 × 5 mL), DCM (3 × 5 mL), and then dried from residue of solvents.

After that, a mixture of DCM/TFA (99.25%/0.75%, 6.5 mL) was added to the resin and stirred for 15 min, then the solution was filtered off from the resin. The solvent was removed under reduced pressure and the residue was re-evaporated three times with DCM. The product was purified by column chromatography (Puriflash, column of PF-15C18AQ-F0025 (15μ 40g), eluent: H_2_O(80%)/MeCN(20%) => H_2_O(0%)/MeCN (100%) for 15 min after MeCN (100%) for 5 min. Compound **16** was obtained as a colorless oil (338 mg, 76% yield).

^1^H-NMR (400 MHz, DMSO-*d_6_*, δ): 12.53 (br.s., 1H, K7COOH), 8.18 (d, *J* = 7.5 Hz, 2H, F5NH + F6NH), 7.99–7.90 (m, 1H, K7NH), 7.89 (t, *J* = 5.2 Hz, *m*) & 7.86 (t, *J* = 5.2 Hz, *n*) (1H, X3NHk, *m* + *n*, *m*/*n* = 3/2), 7.42–7.08 (m, 14H, Ph + Ph + X9H), 6.79 (t, *J* = 5.1 Hz, 1H,K7NHk), 6.35–6.22 (m, 2H, K2NH + E1NH, *m* + *n*), 4.60–4.48 (m, F6Ha + X9Ha(*n*)), 4.48 (s, X9Ha(*m*), *m* + *n, m*/*n* = 3/2), 4.40–4.30 (m, 1H, F5Ha), 4.20–4.09 (m, 1H, K7Ha), 4.08–4.00 (m, 1H, E1Ha), 4.00–3.90 (m, 1H, K2Ha), 3.22 (t, *J* = 7.3 Hz, *n*) & 3.17 (t, *J* = 7.3 Hz, *m*) (2H, K2He, *m* + *n*, *m*/*n* = 3/2), 3.14–3.06 (m, 1H, F6Hb(a)), 3.05–2.82 (m, 6H, F6Hb(b) + X3He + K7He + F5Hb(a)), 2.70–2.57 (m, 1H, F5Hb(b)), 2.37–2.11 (m, 8H, X4Hb + E1Hg + X4Ha + X3Ha), 1.91–1.80 (m, 1H, E1Hb(a)), 1.77–1.12 (m, 19H, E1Hb(b) + K7Hb(a) + K2Hb(a) + K7Hb(b) + K2Hb(b) + X3Hb + X3Hd + K2Hd + K2Hg + X3Hg, *m* + *n*), 1.41–1.32 (m, 36H, tBu).

^13^C-NMR (100 MHz, DMSO-*d_6_*, δ): 173.35 (K7C), 172.24 (K2C(*n*)), 172.20 (K2C(*m*)), 172.14 (X3C(*n*)+ F6C(*m*)), 172.12 (X3C(*m*)), 172.08 (F6C(*n*)), 171.92 (E1C), 171.45 (E1Cd), 171.39 (X4Cg(*mn*)), 171.07 (F5C(*mn*)), 171.00 (X4C(*mn*)), 157.14 (U(*m*)), 157.12 (U(*n*)), 155.60 (K7Boc), 141.17 (X9Cb(*m*)), 140.77 (X9Cb(*n*)),138.13 (F6Cg), 137.99 (F5Cg), 133.43 (X9Ce(*n*)), 133.07 (X9Ce(*m*)), 130.60 (X9Cd(*n*)), 130.27 (X9Cd(*m*)), 129.18 (F6Cd), 129.07 (F5Cd), 128.09 (F6Ce), 128.03 (F5Ce), 127.21 (X9Ct(*m*)), 127.15 (X9Ck(*n*)), 126.86 (X9Ck(*m*)), 126.31 (F6Ck), 126.25 (F5Ck), 126.19 (X9Ct(*n*)),126.06 (X9Cg(*m*)), 124.95 (X9Cg(*n*)), 80.58 (E1tBu), 80.41 (K2tBu(*m*)), 80.32 (K2tBu(*n*)), 79.77 (E1dtBu), 77.37 (K7BoctBu), 54.43 (F5Ca), 53.79 (F6Ca(*m*)), 52.99 (K2Ca(*n*)), 52.86 (K2Ca(*m*)), 52.18 (E1Ca), 52.00 (K7Ca), 49.60 (X9Ca(*n*)), 47.09 (X9Ca(*m*)), 46.79 (K2Ce(*m*)), 45.20 (K2Ce(*n*)), 39.10 (K7Ce(*mn*), 38.61 (X3Ce(*m*)), 38.55 (X3Ce(*n*)), 37.07 (F5Cb), 36.95 (F6Cb), 32.32 (X3Ca(*n*)), 31.95 (X3Ca(*m*)), 31.82 (K2Cb), 30.91 (E1Cg), 30.77 (X4Ca + K7Cb), 30.63 (X4Cb), 29.19 (K7Cd), 29.09 (X3Cd(*m*)), 28.99 (X3Cd(*n*)), 28.29 (tBuK7), 27.75 (tBuE1), 27.66 (tBuK2+ K2Cd(*m*)), 27.63 (tBuE1d+ E1Cb), 26.71 (K2Cd(*n*)), 26.31 (X3Cg(*m*)), 26.22 (X3Cg(*n*)), 24.75 (X3Cb(*m*)), 24.60 (X3Cb(*n*)), 22.73 (K7Cg), 22.44 (K2Cg(*n*)) 22.26 (K2Cg(*m*)).

ESI-MS C_70_H_103_ClN_8_O_16_: *m*/*z* calcd. for [M + H^+^]^+^:1347.72, found:1347.55.

HRMS (m/z, ESI): calcd for C_70_H_103_ClN_8_O_16_-[M + H]^+^1347.7253, found: 1347.7236, 1369.7073 [M + Na]^+^, 1385.6801 [M + K]^+^.

**Compound 11. Scheme 2. Method 2.** To a solution of compound **6** (1 eq.; 245 mg; 0.257 mmol) in DMF (15 mL), DIPEA (1.5 eq.; 66 μL; 0.385 mmol), HOBt(Cl) (1.2 eq.; 44 mg; 0.308 mmol), and HBTU (1.2 eq.; 97 mg; 0.308 mmol) were added, then the resulting mixture was purged with Ar and stirred for 120 min, then compound **10** (1 equiv; 160 mg; 0.257 mmol) was added and the mixture was stirred for 24 h under Ar atmosphere. Then, the solvent was removed under reduced pressure and the residue was dissolved in DCM (25 mL), then extraction was carried out: (1) H_2_O (2 × 30 mL), (2) brine (2 × 30 mL). Then, the organic fraction was dried over Na_2_SO_4_. After the solvent was removed under reduced pressure, the residue was purified by column chromatography (Puriflash 15μ 25g, eluent: DCM(98%)/MeOH(2%) => DCM(92%)/MeOH(8%) for 40 min, where the eluent for TLC was DCM/MeOH = 19:1. As a result, several fractions were obtained with the content of the claimed substance from 21% to 57%. Re-purification was performed using column chromatography (Puriflash on the column PF-15C18HP-F0035 (15μ 35g); eluent: H_2_O(70%)/MeCN(30%) => H_2_O(0%)/MeCN(100%) for 15 min, after MeCN (100%) for 5 min. Compound **12** was obtained as a colorless oil (244 mg, 66% yield).

^1^H-NMR (400 MHz, CDCl_3_, δ): 8.02–7.93 (m, 1H, F5NH), 7.87–7.72 (m, 1H, X3NH(*mn*)), 7.43–6.98 (m, 15H, X9H(mn) + F6NH + Ph + Ph), 6.98–6.88 (m, 1H, X8NH(*mn*), 6.30–6.14 (m, 1H, K7NH(*m* + *n*), 5.52–5.27 (m, 2H, K2NH(*m* + *n*)+ E1NH(*m* + *n*), 5.05–4.90 (m, 1H, K7NHk(*m* + *n*)), 4.60–4.18 (m, 7H, X9Ha(*n*) + F6Ha + X9Ha(*m*) + F5Ha + K7Ha + E1Ha + K2Ha), 3.41–3.00 (m, 10H, X8Hg + K2He + X8Ha + X3He + K7He), 2.97–2.85 (m, 1H, F6Hb(a)), 2.84–2.72 (m, 1H, F6Hb(b)), 2.71–2.59 (m, 1H, F5Hb(a)), 2.47–2.38 (m, 1H, F5Hb(b)), 2.36–2.11 (m, 8H, X4Hb(*mn*) + X4Ha + X3Ha(*mn*) + E1Hg), 2.12–1.97 (m, 1H, E1Hb(a)), 1.91–1.77 (m, 3H, X8Hb + E1Hb(b)), 1.77–1.68 (m, 2H, K7Hb(a) + K2Hb(a)), 1.68–1.10 (m, 16H, K7Hb(b) + K2Hb(b) + X3Hb + X3Hd + K7Hd + K2Hd + K7Hg + K2Hg + X3Hg, *m* + *n*), 1.46–1.38 (m, 36H, tBu).

ESI-MS C_73_H_109_ClN_12_O_15_: *m*/*z* calcd. for [M + H^+^]^+^: 1429.79, found: 1430.60.

**Scheme 3. Method 1.** To a solution of compound **16** (1 eq.; 30 mg; 0.022 mmol) in DMF (3 mL) NH_2_-(CH_2_)_3_-N_3_ (2 eq.; 4 mg; 0.044 mmol), HOBt (1.2 eq.; 4 mg; 0.0264 mmol), HBTU (1.2 eq.; 10 mg; 0.0264 mmol), and DIPEA (1.5 eq.; 6 μL; 0.033 mmol) were added. The mixture was stirred for 24 h in an inert atmosphere. Then, the solvent was removed under reduced pressure and was twice reevaporated with DCM. The residue was purified by column chromatography (Puriflash on column PF-15C18AQ-F0004 (15μ 4g)); eluent: system H_2_O(80%)/MeCN(20%) => H_2_O(0%)/MeCN (100%) for 10 min, after MeCN (100%) for 5 min. Compound **11** was obtained as a colorless oil (21 mg, yield 67%).

^1^H-NMR (400 MHz, DMSO-*d_6_*, δ): 8.34 (d, *J* = 7.2 Hz, 1H, F5NH), 8.19 (d, *J* = 7.5 Hz, 1H, F6NH), 8.01–7.89 (m, 1H, X3NHk(*mn*), *m*/*n* = 3/2), 7.77–7.67 (m, 1H, K7NH(*mn*)), 7.65–7.56 (m, 1H, X8NH(*mn*)), 7.42–7.09 (m, 14H, Ph + Ph + X9H(*mn*)), 6.79 (t, *J* = 5.1 Hz, 1H,K7NHk), 6.34–6.21 (m, K2NH(*mn*) + E1NH(*mn*)), 4.59–4.39 (m, 3H, X9Ha(*n*) + X9Ha(*m*) + F6Ha), 4.37–4.25 (m, 1H, F5Ha), 4.17–4.07 (m, 1H, K7Ha), 4.05–4.00 (m, 1H, E1Ha), 4.00–3.91 (m, 1H, K2Ha), 3.33 (t, *J* = 6.9 Hz, 2H, X8Hg), 3.21 (t, *J* = 7.3 Hz, *n*) & 3.16 (t, *J* = 7.3 Hz, *m*) (2H, K2He, *m* + *n*, *m*/*n = 3*/*2*), 3.14–2.81 (m, 9H, X8Ha + F6Hb(a) + F6Hb(b) + X3He(*mn*) + K7He + F5Hb(a)), 2.71–2.60 (m, 1H, F5Hb(b)), 2.38–2.12 (m, 8H, X4Hb + E1Hg + X4Ha + X3Ha), 1.93–1.80 (m, 1H, E1Hb(a)), 1.72–1.60 (m, 4H, E1Hb(b) + X8Hb + K7Hb(a)), 1.60–1.10 (m, 17H, K2Hb(a) + K7Hb(b) + K2Hb(b) + X3Hb + X3Hd + K2Hd + K2Hg + X3Hg, m + n), 1.41–1.32 (m, 36H, tBu).

ESI-MS C_73_H_109_ClN_12_O_15_: *m*/*z* calcd. for[M − H^+^]^−^: 1429.79, found: 1430.70.

HRMS (*m*/*z*, ESI): calcd. for C_73_H_109_ClN_12_O_15_-[M + Na]^+^: 1451.7748, found: 1451.7716.

**Compound 12. Scheme 2.** Compound **11** (1 eq.; 243 mg; 0.17 mmol) was dissolved in mixture of DCM/TFA (9 mL of DCM, 1 mL of TFA). The mixture was stirred for 12 h, then the solvent was removed under reduced pressure and re-evaporated with DCM three times. The product was precipitated with Et_2_O and washed twice with Et_2_O (10 mL). After, the residue was purified by column chromatography (Puriflash on a column of PF-15C18AQ-F0025 (15μ 25g), eluent: H_2_O(80%)/MeCN(20%) => H_2_O(0%)/MeCN(100%) for 15 min after MeCN (100%) for 5 min. Compound **12** was obtained as a colorless oil (166 mg, yield 84%).

^1^H-NMR (400 MHz, DMSO-*d_6_*, δ): 8.76–8.68 (m, F5NH(*m*)), 8.60–8.53 (m, F5NH(*n*)), 8.54–8.43 (m, F6NH(*m*) + X3NHk(*m*)), 8.42–8.37 (m, F6NH(*n*)), 8.36–8.27 (m, X3NHk(*n*), *m*/*n* = 3/2), 7.76–7.62 (m, 1H, K7NH(*mn*)), 7.59–7.46 (m, 1H, X8NH(*n*) + X8NH(*m*)), 7.43–7.07 (m, 14H, Ph + Ph + X9H(*mn*)) 6.43–6.23 (m, K2NH(*mn*) + E1NH(*mn*)), 4.59–4.44 (m, 2H, X9Ha(*n*) + X9Ha(*m*)), 4.43–4.33 (m, 1H, F6Ha), 4.26–4.16 (m, 1H, F5Ha), 4.16–4.05 (m, 1H, K7Ha), 4.04–3.91 (m, 2H, E1Ha + K2Ha), 3.33 (t, *J* = 6,9 Hz, 2H, X8Hg), 3.25–2.95 (m, 8H, K2He(*mn*) + X8Ha + F6Hb(a) + F6Hb(b) + X3He(*mn*), 2.94–2.82 (m, 1H, F5H(a)), 2.73 (t, *J* = 7.5 Hz, 2H, K7He), 2.70–2.61 (m, 1H, F5Hb(b)), 2.44–2.26 (m, X4Hb(*mn*) + X4Ha(a) + X3Ha(*m*)), 2.25–2.11 (m, E1Hg + X3Ha(*n*) + X4Ha(b)), 1.84-1.69 (m, 3H, E1Hb(a) + E1Hb(b) + K7Hb(a)), 1.69–1.60 (m, 3H, X8Hb + K2Hb(a)), 1.60–1.31 (m, 10H, K7Hb(b) + K2Hb(b) + K7Hd + X3Hb + X3Hd + K2Hd), 1.30–1.12 (m, 6H, K2Hg + K7Hg + X3Hg).

^13^C-NMR (100 MHz, DMSO-*d_6_*, δ): 175.56 (K2C(*m*)), 175.32 (K2C(*n*)), 175.06 (E1C(*m*)), 174.99 (E1C(*n*)), 174.62 (E1Cd(*mn*)), 173.66 (X4Cg(*m*)), 173.40 (X4Cg(*n*)), 172.32 (F5C(*mn*)), 172.23 (X3C(*m*)), 172.13 (X3C(*n*)), 171.97 (X4C(*m*)), 171.84 (X4C(*n*)), 171.26 (K7C(*mn*)), 171.16 (F6C(*m*)), 171.08 (F6C(*n*)), 157.38 (U(*mn*)), 141.22 (X9Cb(m)), 140.79 (X9Cb(n)), 138.16 (F6Cg(*m*)), 138.08 (F6Cg(*n*)), 137.89 (F5Cg(*mn*)), 133.40 (X9Ce(n)), 133.04 (X9Ce(m)), 130.61 (X9Cd(n)), 130.24 (X9Cd(m)), 129.00 (F6Cd), 128.93 (F5Cd), 128.27 (F6Ce), 128.14 (F5Ce), 127.21 (X9Ct(m)), 127.13 (X9Ck(n)), 126.84 (X9Ck(m)), 126.38 (F6Ck), 126.34 (F5Ck), 126.28 (X9Ct(n)), 126.09 (X9Cg(m)), 125.00 (X9Cg(n)), 55.97 (F5Ca(*m*)), 55.65 (F5Ca(*n*)), 55.27 (F6Ca(*m*)), 55.07 (F6Ca(*n*)), 53.17 (K2Ca(*m*)), 52.88 (K2Ca(*n*) + E1Ca(*mn*) + K7Ca(*mn*)), 49.54 (X9Ca(*n*)), 48.20 (X8Cg), 47.05 (X9Ca(*m*)), 46.94 (K2Ce(*m*)), 44.89 (K2Ce(*n*)), 38.76 (X3Ce(*m*)), 38.68 (X3Ce(*n*)), 38.50 (K7Ce(*mn*), 36.62 (F5Cb(*mn*)), 36.25 (F6Cb(*n*)), 36.10 (F6Cb(*m*)), 35.87 (X8Ca), 32.51 (K2Cb(*m*)), 32.28 (K2Cb(*n*) + X3Ca(*n*)), 31.76 (X3Ca(*m*)), 31.17 (K7Cb + E1Cg), 30.72 (X4Ca), 30.46 (X4Cb), 28.93 (E1Cb + X3Cd(*n*)), 28.73 (X3Cd(*m*)), 28.20 (X8Cb), 27.93 (K2Cd(*m*)), 26.89 (K2Cd(*n*) + K7Cd(*mn*)), 26.13 (X3Cg(*mn*)), 24.63 (X3Cb(*mn*)), 22.66 (K7Cg(*m*)), 22.58 (K7Cg(*n*)), 22.40 (K2Cg(*n*)), 22.33 (K2Cg(*m*)).

ESI-MS C_56_H_77_ClN_12_O_13_: *m*/*z* calcd. for [M + H^+^]^+^:1161.55, found: 1161.55.

**Scheme 3. 11** (1 eq.; 39 mg; 0.027 mmol) was dissolved in the system of DCM/TFA/TIPS/H_2_O (46.25%/46.25%/2.5%/5%; *V*/*V* respectively, 2 mL). The mixture was stirred for 3 h, then the solvent was removed under reduced pressure and re-evaporated with DCM three times. The product was precipitated with Et_2_O and washed twice with Et_2_O (1 mL). After, the compound was purified by column chromatography (Puriflash on the column PF-15C18AQ-F0004 (15μ 4g), eluent: H_2_O(80%)/MeCN (20%) => H_2_O(0%)/MeCN (100%) for 15 min, after MeCN (100%) for 5 min. Individual **12** was obtained as a colorless oil (28 mg, yield 88%).

^1^H-NMR (400 MHz, DMSO-*d_6_*, δ): 8.76–8.67 (m, F5NH(*m*)), 8.60–8.53 (m, F5NH(*n*)), 8.52–8.42 (m, F6NH(*m*) + X3NHk(*m*)), 8.42–8.35 (m, F6NH(*n*)), 8.35–8.26 (m, X3NHk(*n*), *m*/*n* = 3/2), 7.75–7.62 (m, 1H, K7NH(*mn*)), 7.59–7.41 (m, 1H, X8NH(*n*) + X8NH(*m*)), 7.43–7.07 (m, 14H, Ph + Ph + X9H(*mn*)) 6.43–6.23 (m, K2NH(*mn*) + E1NH(*mn*)), 4.54–4.44 (m, 2H, X9Ha(*n*) + X9Ha(*m*)), 4.43–4.33 (m, 1H, F6Ha), 4.27–4.17 (m, 1H, F5Ha), 4.17–4.07 (m, 1H, K7Ha), 4.04–3.91 (m, 2H, E1Ha + K2Ha), 3.33 (t, *J* = 6,9 Hz, 2H, X8Hg), 3.25–2.95 (m, 8H, K2He(*mn*) + X8Ha + F6Hb(a) + F6Hb(b) + X3He(*mn*), 2.94–2.85 (m, 1H, F5H(a)), 2.73 (t, *J* = 7.5 Hz, 2H, K7He), 2.70–2.61 (m, 1H, F5Hb(b)), 2.44–2.26 (m, X4Hb(*mn*) + X4Ha(a) + X3Ha(*m*)), 2.25–2.11 (m, E1Hg + X3Ha(*n*) + X4Ha(b)), 1.84–1.69 (m, 3H, E1Hb(a) + E1Hb(b) + K7Hb(a)), 1.69–1.60 (m, 3H, X8Hb + K2Hb(a)), 1.60–1.31 (m, 10H, K7Hd + K7Hb(b) + K2Hb(b) + X3Hb(m) + X3Hb(n) + X3Hd + K2Hd), 1.30–1.12 (m, 6H, K2Hg + K7Hg + X3Hg).

^13^C-NMR (100 MHz, DMSO-*d_6_*, δ): 175.62 (K2C(*m*)), 175.46 (K2C(*n*)), 175.22 (E1C(*m*)), 175.15 (E1C(*n*)), 174.75 (E1Cd(*m*)), 174.71 (E1Cd(*n*)), 173.54 (X4Cg(*m*)), 173.33 (X4Cg(*n*)), 172.30 (F5C(*mn*) + X3C(*m*)), 172.17 (X3C(*n*)), 171.99 (X4C(*m*)), 171.89 (X4C(*n*)), 171.39 (K7C(*mn*)), 171.23 (F6C(*m*)), 171.18 (F6C(*n*)), 157.51 (U(*mn*)), 141.26 (X9Cb(m)), 140.84 (X9Cb(n)), 138.18 (F6Cg(*m*)), 138.12 (F6Cg(*n*)), 137.96 (F5Cg(*mn*)), 133.50 (X9Ce(n)), 133.14 (X9Ce(m)), 130.65 (X9Cd(n)), 130.29 (X9Cd(m)), 129.11 (F6Cd), 129.03 (F5Cd), 128.31 (F6Ce), 128.20 (F5Ce), 127.26 (X9Ct(m)), 127.20 (X9Ck(n)), 126.90 (X9Ck(m)), 126.40 (F6Ck+ F5Ck), 126.32 (X9Ct(n)), 126.14 (X9Cg(m)), 125.03 (X9Cg(n)), 55.83 (F5Ca(*m*)), 55.60 (F5Ca(*n*)), 55.24 (F6Ca(*m*)), 55.10 (F6Ca(*n*)), 53.20 (K2Ca(*m*)), 52.95 (K2Ca(*n*) + E1Ca(*mn*) + K7Ca(*mn*)), 49.72 (X9Ca(*n*)), 48.29 (X8Cg), 47.19 (X9Ca(*m*)), 47.07 (K2Ce(*m*)), 45.26 (K2Ce(*n*)), 38.79 (X3Ce(*m*)), 38.69 (X3Ce(*n*)), 38.62 (K7Ce(*mn*), 36.78 (F5Cb(*mn*)), 36.42 (F6Cb(*n*)), 36.31 (F6Cb(*m*)), 35.95 (X8Ca), 32.55 (K2Cb(*m*)), 32.38 (K2Cb(*n*) + X3Ca(*n*)), 31.90 (X3Ca(*m*)), 31.23 (K7Cb), 31.05 (E1Cg), 30.81 (X4Ca), 30.58 (X4Cb), 28.96 (E1Cb), 28.90 (X3Cd), 28.29 (X8Cb), 28.05 (K2Cd(*m*)), 26.91 (K2Cd(*n*) + K7Cd(*mn*)), 26.28 (X3Cg(*mn*)), 24.76 (X3Cb(*m*)), 24.68 (X3Cb(*n*)), 22.68 (K7Cg(*m*)), 22.58 (K7Cg(*n*)), 22.53 (K2Cg(*n*)) 22.45 (K2Cg(*m*)).

ESI-MS C_56_H_77_ClN_12_O_13_: *m*/*z* calcd. for [M − H^+^]^−^:1161.55, found:1161.55.

HRMS (*m*/*z*, ESI): calcd. for C_56_H_77_ClN_12_O_13_-[M + H]^+^ 1161.5494, found: 1161,5505.

**Compound 17**. A solution of docetaxel (1 eq.; 500 mg; 0.619 mmol), hex-5-ynoic acid (1.1 eq.; 76 mg; 0.68 mmol), and DMAP (0.1 eq.; 7 mg; 0.062 mmol) in DCM was cooled to 0 °C. DIC (1.5 eq.; 117 mg; 0.928 mmol)) was then added dropwise. The reaction mixture was stirred for 4 h at 0 °C and then stirred at room temperature overnight. The solvent was evaporated under reduced pressure. The crude product was purified by chromatography ((Puriflash on column PF-15C18HP-F0040 (15μ 40g), eluent: Hex(95%)/EtOAc(5%) => Hex(0%)/EtOAc(100%) for 25 min after EtOAc (100%) for 5 min.). Compound **17** was obtained as a white crystalline powder (335 mg, yield 60%).

^1^H-NMR (400 MHz, DMSO-*d_6_*, δ): 7.99 (d, *J* = 7.2 Hz, 2H, 25 + 29), 7.89 (d, *J* = 8.9 Hz, 1H, NHBoc), 7.73 (t, *J* = 7.3 Hz, 1H, 27), 7.65 (t, *J* = 7.5 Hz, 2H, 26 + 28), 7.42 (t, *J* = 7.5 Hz, 2H, 35 + 37), 7.39–7.32 (m, 2H, 34 + 38), 7.17 (t, *J* = 7.2 Hz, 1H, 36), 5.83–5.70 (m, 1H, 13), 5.40 (d, *J* = 6.5 Hz, 1H, 2), 5.14–5.04 (m, 3H, 31 + 10 + 32), 5.02 (d, *J* = 7.2 Hz, 1H, 7OH), 4.93 (d, *J* = 2.5 Hz, 1H, 10OH), 4.92–4.87 (m, 1H, 5), 4.45(br.s, 1H, 1OH), 4.10–3.98 (m, 3H, 7 + 20a + 20b), 3.63 (d, *J* = 6.2 Hz, 1H, 3), 2.83 (t, *J* = 2.6 Hz, 1H, X10Hk), 2.55–2.50 (m, 2H, X10Hb), 2.32–2.25 (m, 1H, 6Hb), 2.24 (s, 3H, 22), 2.23–2.16 (m, 2H, X10Hd), 1.87–1.78 (m, 1H, 14Hb), 1.77–1.71 (m, 2H, X10Hg), 1.69 (s, 3H, 18), 1.67–1.59 (m, 1H, 6Ha), 1.55–1.46 (m, 4H, 19 + 14Ha), 1.38 (s, 9H, tBu), 0.98 (s, 6H, 16 + 17).

^13^C-NMR (100 MHz, DMSO-*d_6_*, δ): 209.35 (9), 171.81 (X10Ca), 169.61 (21), 169.11 (30), 165.32 (23), 155.21 (C(O)Boc), 137.49 (33), 136.93 (11), 135.93 (12), 133.46 (27), 130.06 (24), 129.60 (25/29), 128.71 (26/28), 128.61 (35/37), 128.10 (36), 127.46 (34/38), 83.80 (5), 83.51 (X10Ce), 80.30 (4), 78.52 (CBoc), 76.81 (1), 75.43 (20), 75.11 (31), 74.81 (2), 73.76 (10), 71.91 (X10Ck), 71.21 (13), 70.77 (7), 57.00 (8), 55.14 (32), 45.98 (3), 42.91 (15), 36.50 (6), 34.71 (14), 32.10 (X10Cb), 28.16 (tBu), 26.46 (16), 23.47 (X10Cg), 22.53 (22), 20.79 (17), 17.03 (X10Cd), 13.68 (18), 9.83 (19).

HRMS (*m*/*z*, ESI): calcd. for C_49_H_59_N_15_O_13_-[M + H]^+^ 902.3957, found: 902.3981.

**Compound 18**. Compounds **12** (1 eq.; 162 mg; 0.127 mmol) and **17** (1 eq.; 115 mg; 0.127 mmol), CuSO_4_·5H_2_O (0.4 eq.; 13 mg; 0.05 mmol) were dissolved in DMF/H_2_O (6 mL/1 mL). After, the system was purged with argon. To the mixture, sodium ascorbate (1.2 eq.; 30 mg; 0.152 mmol) was added in H_2_O (1 mL) with a syringe. The resulting solution was stirred for 24 h in an inert atmosphere. After, EDTA (0.8 eq.; 30 mg; 0.1 mmol) was added. The mixture was stirred for 3 h. After the reaction, the mixture was filtered from the precipitate and the solvent was removed under reduced pressure. The product was precipitated with MeCN and washed twice with MeCN (2 mL). After, the residue was purified by column chromatography (Puriflash on a column of PF-15C18AQ-F0025 (15μ 25g), eluent: H_2_O(90%)/MeCN(10%) => H_2_O(0%)/MeCN(100%) for 20 min after MeCN (100%) for 5 min. Compound **18** was obtained as a pink powder (99 mg, yield 38%).

^1^H-NMR (600 MHz, DMSO-*d_6_*, δ): 8.70-8.62 (m, F5NH(*m*)), 8.55-8.48 (m, F5NH(*n*)), 8.48–8.43 (m, F6NH(*m*)), 8.43–8.37 (X3NHk(*m*)), 8.37–8.31 (m, F6NH(*n*)), 8.28–8.20 (m, X3NHk(*n*)), 7.98 (d, *J* = 7.2 Hz, 2H, 25 + 29), 7.87 (d, *J* = 8.9 Hz, 1H, NHBoc), 7.83 (s, 1H, X10Hk), 7.76–7.68 (m, 2H, 27 + K7NH(*mn*)), 7.68–7.57 (m, 3H, 26 + 28 + X8NH(*n*) + X8NH(*m*)), 7.40 (t, *J* = 7.5 Hz, 2H, 35 + 37), 7.38–7.34 (m, 2H, 34 + 38), 7.35–7.06 (m, 15H, Ph + Ph + X9H(*mn*) + 36), 6.43–6.20 (m, 2H, K2NH(*mn*) + E1NH(*mn*)), 5.82–5.72 (m, 1H, 13), 5.39 (d, *J* = 6.5 Hz, 1H, 2), 5.13–5.00 (m, 3H, 31 + 10 + 32), 4.94 (br.s., 1H, OH), 4.90 (d, *J* = 9.4 Hz, 1H, 5), 4.57–4.47 (m, 2H, X9Ha(*n*) + X9Ha(*m*)), 4.45-4.37 (m, 2H, OH + F6Ha), 4.30 (t, *J* = 6.9 Hz,2H, X8Hg), 4.27-4.18 (m, 1H, F5Ha), 4.18–4.10 (m, 1H, K7Ha), 4.08–3.94 (m, 5H, 7 + 20a + E1Ha + 20b + K2Ha), 3.63 (d, *J* = 6.2 Hz, 1H, 3), 3.22–2.95 (m, 8H, K2He(*mn*) + F6Hb(a) + X8Ha +F6Hb(b) + X3He(*mn*)), 2.93–2.84 (m, 1H, F5Hb(a)), 2.74 (t, *J* = 7.5 Hz, 2H, K7He), 2.71–2.65 (m, 1H, F5Hb(b)), 2.62 (t, J = 7.0 Hz, 2H, X10Hd), 2.45 (t, J = 7.0 Hz, 2H, X10Hb), 2.42–2.11 (m, 9H, X4Hb(*mn*) + X4Ha(a) + X3Ha(*m*) + 6Hb + E1Hg + X3Ha(n) + X4Ha(b)), 2.23 (s. 3H, 22), 1.99–1.90 (m, 2H, X8Hb), 1.90–1.84 (m, 2H, X10Hg), 1.84–1.69 (m, 4H, 14Hb + E1Hb(a) + E1Hb(b) + K7Hb(a)), 1.70 (s. 3H, 18), 1.68–1.58 (m, 3H, 14Ha + 6Ha + K2Hb(a)), 1.58–1.31 (m, 10H, K7Hd + K7Hb(b) + K2Hb(b) + X3Hb(m) + X3Hb(n) +X3Hd(mn) + K2Hd(mn)), 1.50 (s. 3H, 19), 1.33 (s. 9H, tBu), 1.30–1.16 (m, 6H, K2Hg + K7Hg + X3Hg), 0.97 (s. 6H, 16 + 17).

^13^C-NMR (100 MHz, DMSO-*d_6_*, δ): 209.31 (9), 175.25 (K2C(*m*)), 175.21 (K2C(*n*)), 174.81 (E1C(*m*)), 174.76 (E1C(*n*)), 174.34 (E1Cd(*m*)), 174.25 (E1Cd(*n*)), 173.59 (X4Cg(*mn*)), 172.30 (F5C(*mn*)), 172.21 X3C(*mn*)), 172.07 (X10Ca + X3C(*n*)), 171.92 (X4C(*m*)), 171.87 (X4C(*n*)), 171.33 (K7C(*mn*)), 171.15 (F6C(*m*)), 171.10 (F6C(*n*)), 169.57 (21), 169.11 (30), 165.29 (23), 157.30 (U(*mn*)), 155.19 (C(O)Boc), 145.87 (X10Ce), 141.19 (X9Cb(*m*)), 140.76 (X9Cb(n)), 138.10 (F6Cg(*m*)), 138.02 (F6Cg(*n*)), 137.78 (F5Cg(*mn*)), 137.50 (33), 136.89 (11), 135.93 (12), 133.43 (27), 133.37 (X9Ce(n)), 133.01 (X9Ce(m)), 130.59 (X9Cd(n)), 130.22 (X9Cd(m)), 130.03 (24), 129.56 (25/29), 128.97 (F6Cd), 128.89 (F5Cd), 128.68 (26/28), 128.57 (35/37), 128.23 (F6Ce), 128.11 (F5Ce), 128.06 (36), 127.41 (34/38), 127.20 (X9Ct(m)), 127.15 (X9Ck(n)), 126.82 (X9Ck(m)), 126.36 (F6Ck), 126.30 (X9Ct(n) + F5Ck), 126.08 (X9Cg(m)), 124.99 (X9Cg(n)), 122.03 (X10Ck), 83.76 (5), 80.27 (4), 78.46 (CBoc), 76.80 (1), 75.40 (20), 75.01 (31), 74.79 (2), 73.72 (10), 71.16 (13), 70.74 (7), 56.97 (8), 55.89 (F5Ca(*mn*)), 55.20 F6Ca(*mn*)), 55.11 (32), 52.85 (K2Ca(*mn*) + E1Ca(*mn*)), 52.65 (K7Ca(*mn*)), 49.60 (X9Ca(*n*)), 47.00 (X9Ca(*m*)), 46.85 (X8Cg+ K2Ce(*m*)), 45.96 (3), 45.31 (K2Ce(*n*)), 42.88 (15), 38.71 (X3Ce(*m*)), 38.61 (X3Ce(*n*)), 38.50 (K7Ce(*mn*), 36.66 (F5Cb(*mn*)), 36.47 (6 + F6Cb(*mn*)), 35.82 (X8Ca), 34.69 (14), 32.65 (X10Cb), 32.30 (K2Cb(*m*)), 32.25 (K2Cb(*n*) + X3Ca(*n*)), 31.74 (X3Ca(*m*)), 31.09 (K7Cb), 30.92 (E1Cg), 30.70 (X4Ca), 30.48 (X4Cb), 29.81 (X8Cb), 28.66(E1Cb + X3Cd), 28.08 (tBu), 27.83 (K2Cd(*m*)), 26.85 (K2Cd(*n*) + K7Cd(*mn*)), 26.44 (16), 26.08 (X3Cg(*mn*)), 24.59 (X3Cb(*m*)), 24.56 (X3Cb(*n*)), 24.29 (X10Cg), 24.18 (X10Cd), 22.60 (K7Cg(*mn*)), 22.50 (22), 22.27 (K2Cg(*mn*)), 20.77 (17), 13.65 (18), 9.79 (19).

ESI-MS C_105_H_136_ClN_13_O_28_: *m*/*z* calcd. for [M + 2H^+^]^2+^ 1031.97, found:1032.60.

HRMS (*m*/*z*, ESI): calcd. for C_105_H_136_ClN_13_O_28_- [M + 2H]^2+^ 1031.9726, found: 1031,9761.

**Compound 19**. Compound **18** (1 eq.; 14 mg; 6.43 μmol) and DIPEA (8 eq.; 6.7 mg; 51.4 μmol) were dissolved in DMF (2 mL). After, the system was purged with argon. To the mixture, Sulfo-Cy5 NHS-ester (1 eq.; 5 mg; 6.43 μmol) was added. The mixture was stirred for 6 h. After, the solvent was evaporated under reduced pressure. The product was precipitated with MeCN and washed twice with MeCN (2 mL). After, the residue was purified by column chromatography (Puriflash on a column of PF-15C18AQ-F0025 (15μ 25g), eluent: H_2_O(90%)/MeCN(10%) => H_2_O(0%)/MeCN(100%) for 20 min after MeCN (100%) for 5 min. Compound **19** was obtained as a blue powder (15.8 mg, yield 92%).

^1^H-NMR (600 MHz, DMSO-*d_6_*, δ): 8.41–8.29 (m, 2H, ArSulfoCy5), 8.24–8.17 (m, 1H, ArSulfoCy5), 7.97 (d, *J* = 7.2 Hz, 2H, 25 + 29), 7.87 (d, *J* = 8.9 Hz, 1H, NHBoc), 7.84 (s, 1H, X10Hk), 7.82–7.79 (m, 1H, ArSulfoCy5), 7.79–7.73 (m, 1H, 27), 7.73–7.68 (m, 1H, ArSulfoCy5), 7.68–7.61 (m, 3H, 26 + 28 + ArSulfoCy5), 7.40 (t, *J* = 7.5 Hz, 2H, 35 + 37), 7.37–7.33 (m, 2H, 34 + 38), 7.34–7.06 (m, 16H, Ph + Ph + X9H(*mn*) + 36 + SulfoCy5(C=C)), 6.55 (t, *J* = 12.6 Hz, 1H, SulfoCy5(C=C)), 6.42–6.20 (m, 3H, SulfoCy5(C=C)), 5.82–5.74 (m, 1H, 13), 5.38 (d, *J* = 6.5 Hz, 1H, 2), 5.12–5.00 (m, 3H, 31 + 10 + 32), 4.95 (br.s., 1H, OH), 4.89 (d, *J* = 9.4 Hz, 1H, 5), 4.57–4.44 (m, 2H, X9Ha(*n*) + X9Ha(*m*)), 4.45–4.37 (m, 2H, OH + F6Ha), 4.35–4.23 (m, 3H, X8Hg + F5Ha), 4.17–3.94 (m, 6H, K7Ha + 7+20a + E1Ha + 20b + K2Ha), 3.62 (d, *J* = 6.2 Hz, 1H, 3), 3.57 (s, 3H, 28′), 3.36 (br.s, 1H, OH), 3.53–2.95 (m, 12H, 6′ + K2He(*mn*) + F6Hb(a) + X8Ha+ K7He + F6Hb(b) + X3He(*mn*)), 2.93–2.84 (m, 1H, F5H(a)), 2.69–2.63 (m, 1H, F5Hb(b)), 2.61 (t, *J* = 7.0 Hz, 2H, X10Hd), 2.46–2.40 (m, 2H, X10Hb), 2.37–2.11 (m, 9H, X4Hb(*mn*) + X4Ha(a) + X3Ha(*m*) + 6Hb + E1Hg + X3Ha(n) +X4Ha(b)), 2.22 (s. 3H, 22), 2.01 (t, *J* = 7.0 Hz, 2H, 2′), 1.96–1.90 (m, 2H, X8Hb), 1.90–1.84 (m, 2H, X10Hg), 1.84–1.70 (m, 4H, 14Hb + E1Hb(a) + E1Hb(b) + K7Hb(a)), 1.70–1.62 (m. 15H, 18 + 29′ + 30′ + 31′ + 32′), 1.68–1.58 (m, 3H, 14Ha + 6Ha + K2Hb(a)), 1.58–1.31 (m, 10H, K7Hd + K7Hb(b) + K2Hb(b) + X3Hb(m) + X3Hb(n) +X3Hd(mn) + K2Hd(mn)), 1.49 (s. 3H, 19), 1.32 (s. 9H, tBu), 1.30–1.16 (m, 12H, 5′ + 3′ + 4′ + K2Hg + K7Hg +X3Hg), 0.97 (s. 6H, 16 + 17).

ESI-MS C_137_H_172_ClN_15_O_35_S_2_: *m*/*z* calcd. for [M + 2H^+^]^2+^: 1344.57, found: 1345.05.

HRMS (*m*/*z*, ESI): calcd. for C_137_H_172_ClN_15_O_35_S_2_-[M + 2H^+^]^2+^ 1344.5725, found: 1344.5768.

## 4. Conclusions

Herein, we designed and synthesized a new PSMA-targeting, DCL-based molecular platform **12** for bimodal or theranostic agent delivery to prostate cancer cells. Its conjugate **19** with docetaxel and fluorescent label Sulfo-Cy5 was also synthesized, demonstrating the possibility to stepwise conjugate the proposed vector molecule with two different functional moieties in orthogonal chemical conditions.

Two alternative methods to obtain polypeptide-based compound **12** using liquid- and solid-phase techniques, including 13 to 16 sequential stages, were compared. The optimal method for stereoselective synthesis of molecular platform **12** consists in solid-phase synthesis of a peptide sequence of the linker, coupling of a polypeptide to a DCL vector fragment, subsequent attachment of 3-aminopropylazide under optimized conditions, and final removal of the protective groups.

The obtained compounds were characterized by NMR spectroscopy and high-resolution mass spectrometry; complete assignment of signals in the NMR spectra of the compounds **12** and **18** was made using two-dimensional NMR sequences. The reasonable cytotoxicity of vector molecule **12**, its conjugate with docetaxel **18,** and docetaxel/Sulfo-Cy5 **19** against PSMA-expressing cell lines were found during initial in vitro study as well as their selective interaction with cells. However, further in vitro as far as in vivo investigations of the conjugates are required for a more explicit demonstration of their efficacy and selectivity for PSMA-expressing cells and tumors. Anyhow, conjugate **19** can be used as a convenient starting point appropriate for the follow-up structure optimization study.

## Figures and Tables

**Table 1:** Comparison of synthetical approaches to obtain target compound **12**.

	Liquid-Phase Technique (Scheme 2)	SPPS Technique (Scheme 3)
Yield based on starting Boc-Fmoc-Lysine	25%	45%
Yield based on compound **6**	55%	37%
The total number of synthesis steps (stages with chromatographic separation)	13 (10)	16 (7)

## Supplementary Materials

The following are available online. Figure S1: ^1^H-NMR spectrum of compound **6** in DMSO-*d*_6_; Figure S2: ^13^C-NMR spectrum of compound **6** in DMSO-*d*_6_; Figure S3: ^1^H-NMR spectrum of compound 7 in CDCl_3_; Figure S4: ^1^H-NMR spectrum of compound **10** in DMSO-*d*_6_; Figure S5: ^1^H-NMR spectrum of compound **16** in DMSO-*d*_6_; Figure S6: ^13^C NMR spectrum of compound **16** in DMSO-*d*_6_; Figure S7: ^1^H-NMR spectrum of compound **11** in CDCl_3._ Liquid-phase technique. Method 2; Figure S8: ^1^H-NMR spectrum of compound **11** in DMSO-*d*_6_. SPPS technique. Method 1: Figure S9: ^1^H-NMR spectrum of compound **12** in DMSO-*d*_6_. Liquid-phase technique; Figure S10: ^13^C NMR spectrum of compound **12** in DMSO-*d*_6_. Liquid-phase technique: Figure S11: ^1^H-NMR spectrum of compound **12** in DMSO-*d*_6_. SPPS technique: Figure S12: ^13^C-NMR spectrum of compound **12** in DMSO-*d*_6_. SPPS technique; Figure S13: ^1^H-NMR spectrum of compound **17** in DMSO-*d*_6_; Figure S14: ^13^C-NMR spectrum of compound **17** in DMSO-*d*_6_; Figure S15: HSQC ^1^H-^13^C spectrum of compound **17** in DMSO-*d*_6_, T = 296 K; Figure S16: HMBC ^1^H-^13^C spectrum of compound **17** in DMSO-*d*_6_, T = 296 K; Figure S17: ^1^H-NMR spectrum of compound **18** in DMSO-*d*_6_; Figure S18: ^13^C-NMR spectrum of compound **18** in DMSO-*d*_6_; Figure S19: HSQC ^1^H-^13^C spectrum of compound **18** in DMSO-*d*_6_, T = 296 K; Figure S20: HMBC ^1^H-^13^C spectrum of compound **18** in DMSO-*d*_6_, T = 296 K; Figure S21: ^1^H-NMR spectrum of compound **19** in DMSO-*d*_6_; Table S1: HSQC (^13^CΔδ/^1^HΔδ (ppm/ppm)) of 17 in DMSO-*d*_6_, T = 296 K; Table S2: HMBC (^13^CΔδ/^1^HΔδ (ppm/ppm)) of 17 in DMSO-*d*_6_, T = 296 K; Table S3: HSQC (^13^CΔδ/^1^HΔδ (ppm/ppm)) of 18 in DMSO-*d*_6_, T = 296 K; Table S4: HMBC (^13^CΔδ/^1^HΔδ (ppm/ppm)) of 18 in DMSO-*d*_6_, T = 296 K.

## References

[1] Rahbar K., Afshar-Oromieh A., Jadvar H., Ahmadzadehfar H. (2018). PSMA Theranostics: Current Status and Future Directions. Mol. Imaging.

[2] Bray F., Ferlay J., Soerjomataram I., Siegel R.L., Torre L.A., Jemal A. (2018). Global cancer statistics 2018: GLOBOCAN estimates of incidence and mortality worldwide for 36 cancers in 185 countries. CA Cancer J. Clin..

[3] Wester H.J., Schottelius M. (2019). PSMA-Targeted Radiopharmaceuticals for Imaging and Therapy. Semin. Nucl. Med..

[4] Mottet N., Bellmunt J., Bolla M., Briers E., Cumberbatch M.G., De Santis M., Fossati N., Gross T., Henry A.M., Joniau S. (2017). EAU-ESTRO-SIOG Guidelines on Prostate Cancer. Part 1: Screening, Diagnosis, and Local Treatment with Curative Intent. Eur. Urol..

[5] Edwards B.K., Noone A.M., Mariotto A.B., Simard E.P., Boscoe F.P., Henley S.J., Jemal A., Cho H., Anderson R.N., Kohler B.A. (2014). Annual Report to the Nation on the status of cancer, 1975–2010, featuring prevalence of comorbidity and impact on survival among persons with lung, colorectal, breast, or prostate cancer. Cancer.

[6] Cornford P., Bellmunt J., Bolla M., Briers E., De Santis M., Gross T., Henry A.M., Joniau S., Lam T.B., Mason M.D. (2017). EAU-ESTRO-SIOG Guidelines on Prostate Cancer. Part II: Treatment of Relapsing, Metastatic, and Castration-Resistant Prostate Cancer. Eur. Urol..

[7] Thompson I.M., Tangen C.M. (2014). Prostate cancer screening comes of age. Lancet.

[8] Kumar A., Mastren T., Wang B., Hsieh J.T., Hao G., Sun X. (2016). Design of a Small-Molecule Drug Conjugate for Prostate Cancer Targeted Theranostics. Bioconjug. Chem..

[9] Tan P.S., Aguiar P., Haaland B., Lopes G. (2018). Addition of abiraterone, docetaxel, bisphosphonate, celecoxib or combinations to androgen-deprivation therapy (ADT) for metastatic hormone-sensitive prostate cancer (mHSPC): A network meta-analysis. Prostate Cancer Prostatic Dis..

[10] Sokol M.B., Nikolskaya E.D., Yabbarov N.G., Zenin V.A., Faustova M.R., Belov A.V., Zhunina O.A., Mollaev M.D., Zabolotsky A.I., Tereshchenko O.G. (2019). Development of novel PLGA nanoparticles with co-encapsulation of docetaxel and abiraterone acetate for a highly efficient delivery into tumor cells. J. Biomed. Mater. Res. Part B Appl. Biomater..

[11] Jang J.H., Han S.J., Kim J.Y., Kim K.I., Lee K.C., Kang C.S. (2019). Synthesis and Feasibility Evaluation of a new Trastuzumab Conjugate Integrated with Paclitaxel and 89 Zr for Theranostic Application Against HER2-Expressing Breast Cancers. ChemistryOpen.

[12] Kelkar S.S., Reineke T.M. (2011). Theranostics: Combining imaging and therapy. Bioconjug. Chem..

[13] Opoku-Damoah Y., Wang R., Zhou J., Ding Y. (2016). Versatile nanosystem-based cancer theranostics: Design inspiration and predetermined routing. Theranostics.

[14] Wang H., Byun Y., Barinka C., Pullambhatla M., Bhang H.C., Fox J.J., Lubkowski J., Mease R.C., Pomper M.G. (2010). Bioisosterism of urea-based GCPII inhibitors: Synthesis and structure-activity relationship studies. Bioorg. Med. Chem. Lett..

[15] Hillier S.M., Maresca K.P., Femia F.J., Marquis J.C., Foss C.A., Nguyen N., Zimmerman C.N., Barrett J.A., Eckelman W.C., Pomper M.G. (2009). Preclinical evaluation of novel glutamate-urea-lysine analogues that target prostate-specific membrane antigen as molecular imaging pharmaceuticals for prostate cancer. Cancer Res..

[16] Mesters J.R., Barinka C., Li W., Tsukamoto T., Majer P., Slusher B.S., Konvalinka J., Hilgenfeld R. (2006). Structure of glutamate carboxypeptidase II, a drug target in neuronal damage and prostate cancer. EMBO J..

[17] Machulkin A.E., Ivanenkov Y.A., Aladinskaya A.V., Veselov M.S., Aladinskiy V.A., Beloglazkina E.K., Koteliansky V.E., Shakhbazyan A.G., Sandulenko Y.B., Majouga A.G. (2016). Small-molecule PSMA ligands. Current state, SAR and perspectives. J. Drug Target..

[18] Benešová M., Bauder-Wüst U., Schäfer M., Klika K.D., Mier W., Haberkorn U., Kopka K., Eder M. (2016). Linker Modification Strategies to Control the Prostate-Specific Membrane Antigen (PSMA)-Targeting and Pharmacokinetic Properties of DOTA-Conjugated PSMA Inhibitors. J. Med. Chem..

[19] Ivanenkov Y.A., Machulkin A.E., Garanina A.S., Skvortsov D.A., Uspenskaya A.A., Deyneka E.V., Trofimenko A.V., Beloglazkina E.K., Zyk N.V., Koteliansky V.E. (2019). Synthesis and biological evaluation of Doxorubicin-containing conjugate targeting PSMA. Bioorg. Med. Chem. Lett..

[20] Machulkin A.E., Skvortsov D.A., Ivanenkov Y.A., Ber A.P., Kavalchuk M.V., Aladinskaya A.V., Uspenskaya A.A., Shafikov R.R., Plotnikova E.A., Yakubovskaya R.I. (2019). Synthesis and biological evaluation of PSMA-targeting paclitaxel conjugates. Bioorg. Med. Chem. Lett..

[21] Machulkin A.E., Uspenskaya A.A., Ber A.P., Petrov S.A., Saltykova I.V., Ivanenkov Y.A., Skvortsov D.A., Erofeev A.S., Gorelkin P.V., Beloglazkina E.K. (2019). Peptide Agent Comprising a Urea Derivative Based PSMA-Binding Ligand, a Method for Preparing the Same, and Use for Producing a Conjugate with a Drug and Diagnostic Agent. RF Patent.

[22] Kularatne S.A., Venkatesh C., Santhapuram H.K.R., Wang K., Vaitilingam B., Henne W.A., Low P.S. (2010). Synthesis and biological analysis of prostate-specific membrane antigen-targeted anticancer prodrugs. J. Med. Chem..

[23] Dubowchik G.M., Firestone R.A., Padilla L., Willner D., Hofstead S.J., Mosure K., Knipe J.O., Lasch S.J., Trail P.A. (2002). Cathepsin B-labile dipeptide linkers for lysosomal release of doxorubicin from internalizing immunoconjugates: Model studies of enzymatic drug release and antigen-specific in vitro anticancer activity. Bioconjug. Chem..

[24] Bollhagen R., Schmiedberger M., Barlos K., Grell E. (1994). Chloride Resin. J. Chem. Soc. Chem. Commun..

[25] Behrendt R., White P., Offer J. (2016). Advances in Fmoc solid-phase peptide synthesis. J. Pept. Sci..

[26] Benoiton N.L. (2006). Chemistry of Peptide Synthesis.

[27] Yang Y. (2016). Side Reactions in Peptide Synthesis.

[28] Herbst R.S., Khuri F.R. (2003). Mode of action of docetaxel—A basis for combination with novel anticancer agents. Cancer Treat. Rev..

[29] Guénard D., Guéritte-Voegelein F., Potier P. (1993). Taxol and Taxotere: Discovery, Chemistry, and Structure-Activity Relationships. Acc. Chem. Res..

[30] Tang W., Becker M.L. (2014). “Click” reactions: A versatile toolbox for the synthesis of peptide-conjugates. Chem. Soc. Rev..

[31] Schottelius M., Wurzer A., Wissmiller K., Beck R., Koch M., Gorpas D., Notni J., Buckle T., Van Oosterom M.N., Steiger K. (2019). Synthesis and preclinical characterization of the PSMA-targeted hybrid tracer PSMA-I&F for nuclear and fluorescence imaging of prostate cancer. J. Nucl. Med..

[32] Gorges T.M., Riethdorf S., von Ahsen O., Nastaly P., Röck K., Boede M., Peine S., Kuske A., Schmid E., Kneip C. (2016). Heterogeneous PSMA expression on circulating tumor cells—A potential basis for stratification and monitoring of PSMA-directed therapies in prostate cancer. Oncotarget.

[33] Kruh G.D. (2005). Ins and outs of taxanes. Cancer Biol. Ther..

[34] Tietze L.F., Eicher T. (1999). Preparative Organic Chemistry.

[35] Angeli A., Li M., Dupin L., Vergoten G., Noël M., Madaoui M., Wang S., Meyer A., Géhin T., Vidal S. (2017). Design and Synthesis of Galactosylated Bifurcated Ligands with Nanomolar Affinity for Lectin LecA from Pseudomonas aeruginosa. ChemBioChem.

