# Supplementary material for: Polypeptide-Based Molecular Platform and Its Docetaxel/Sulfo-Cy5-Containing Conjugate for Targeted Delivery to Prostate Specific Membrane Antigen
Source: Molecules. 2020 Dec 8;25(24):5784. doi: 10.3390/molecules25245784 (PMC7762530; doi:10.3390/molecules25245784)
Supplement: Supplementary file 1 [file molecules-25-05784-s001.pdf]

Compound 6

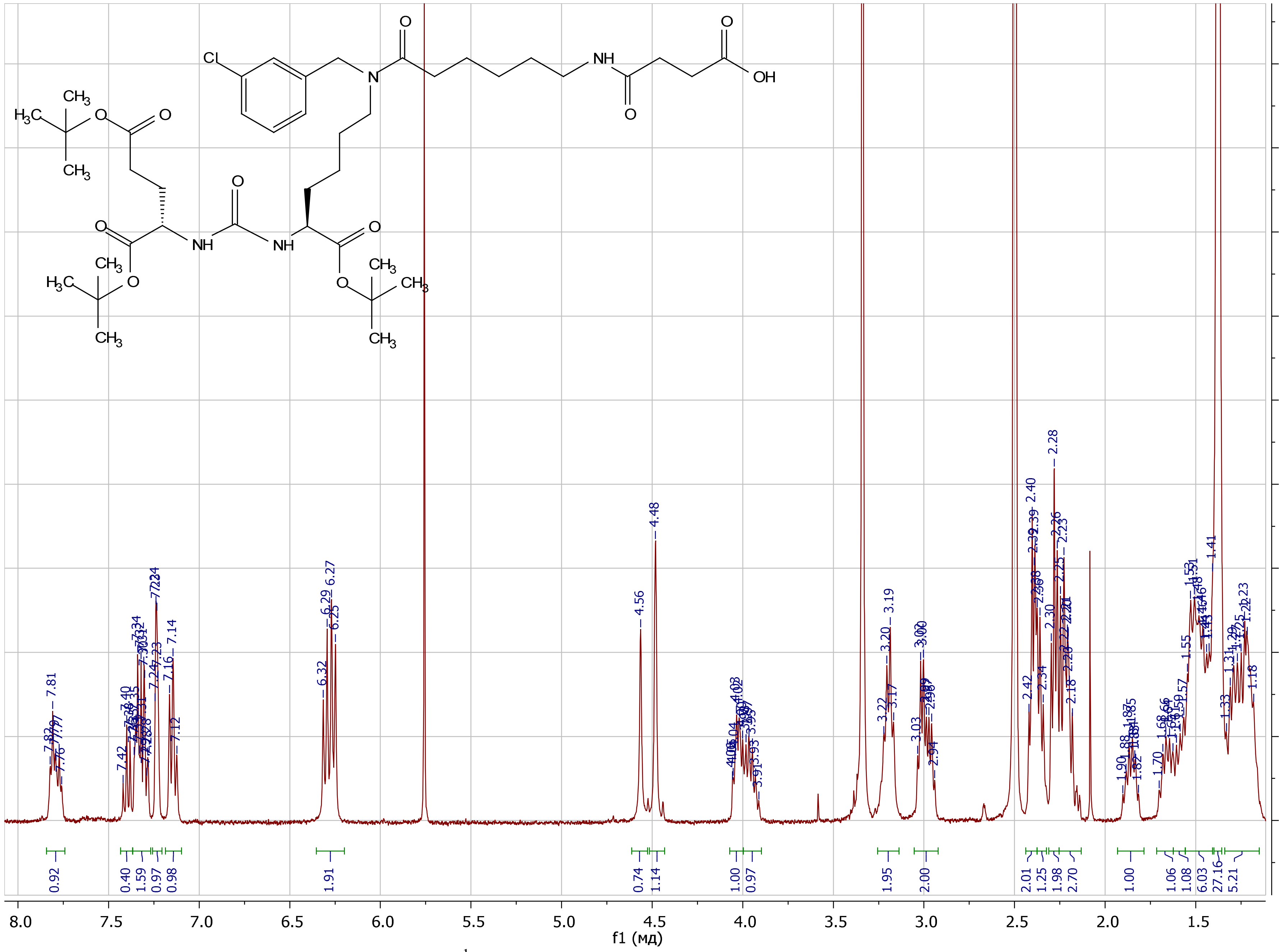

Figure S1. ^{1}H NMR spectrum of compound № **6** in DMSO-d_6.

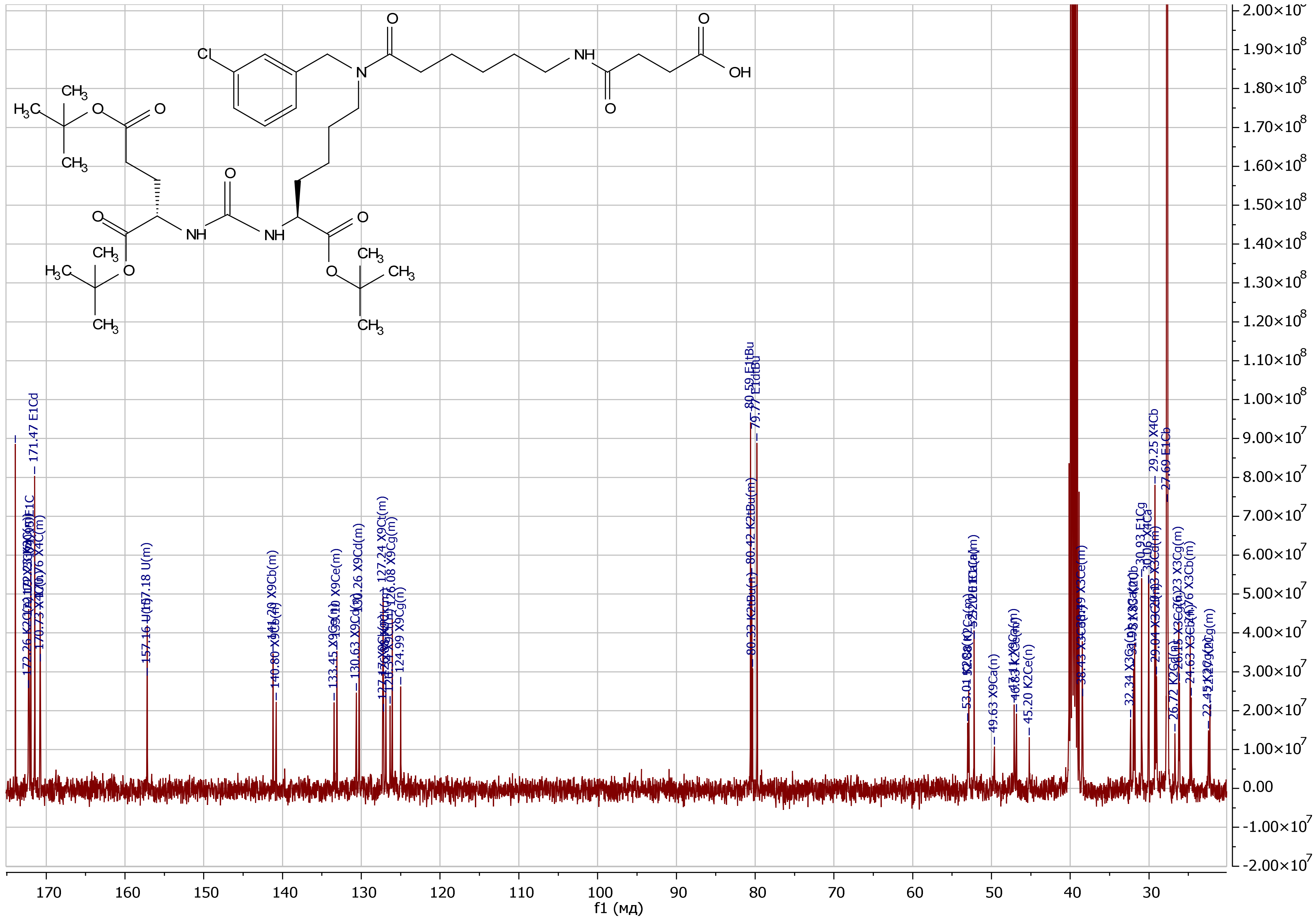

Figure S2. ^{13}C NMR spectrum of compound № **6** in DMSO-d_6.

Compound 7.

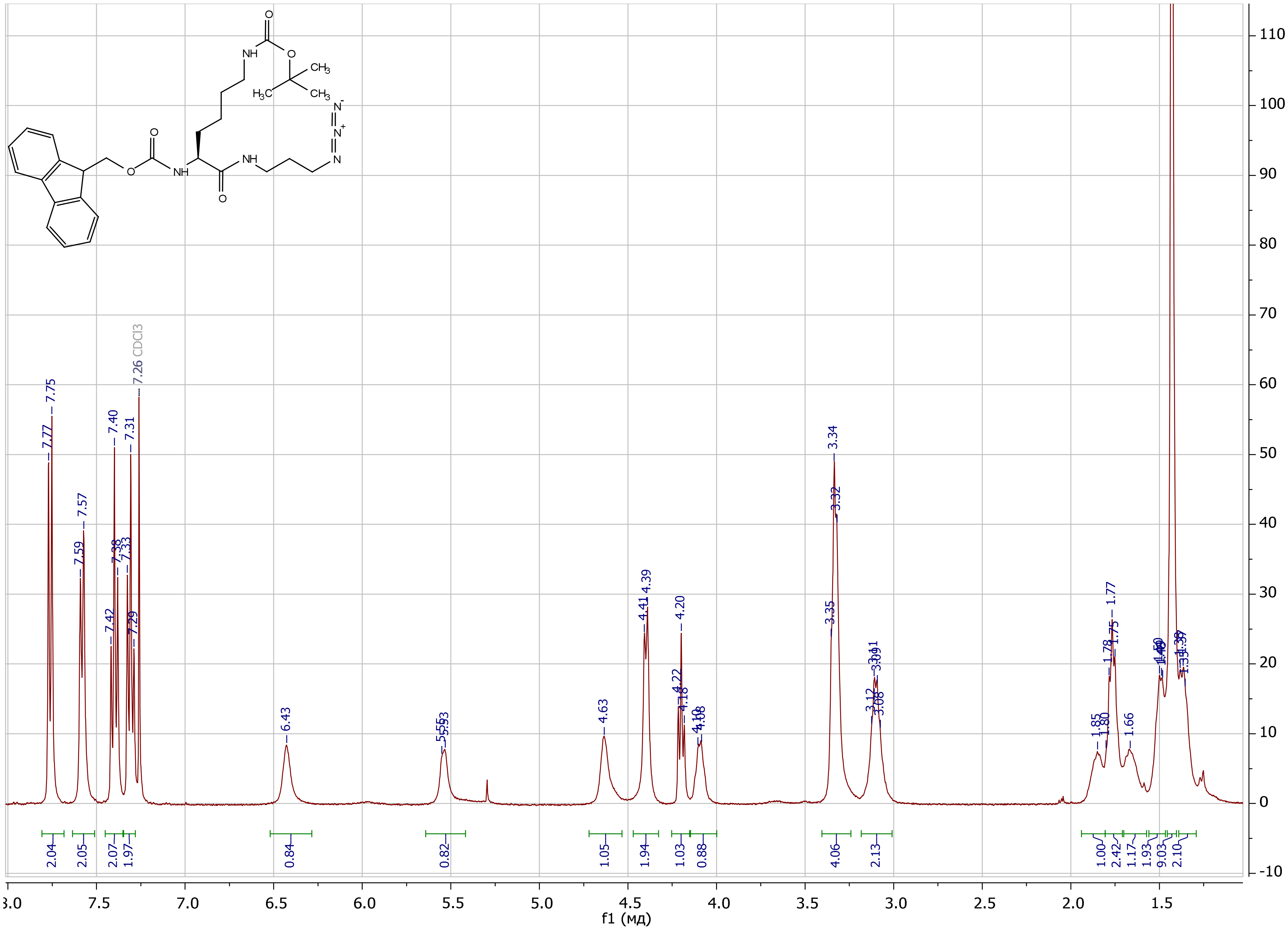

Figure S3. ^{1}H NMR spectrum of compound № **7** in $CDCl_3$.

Compound 10

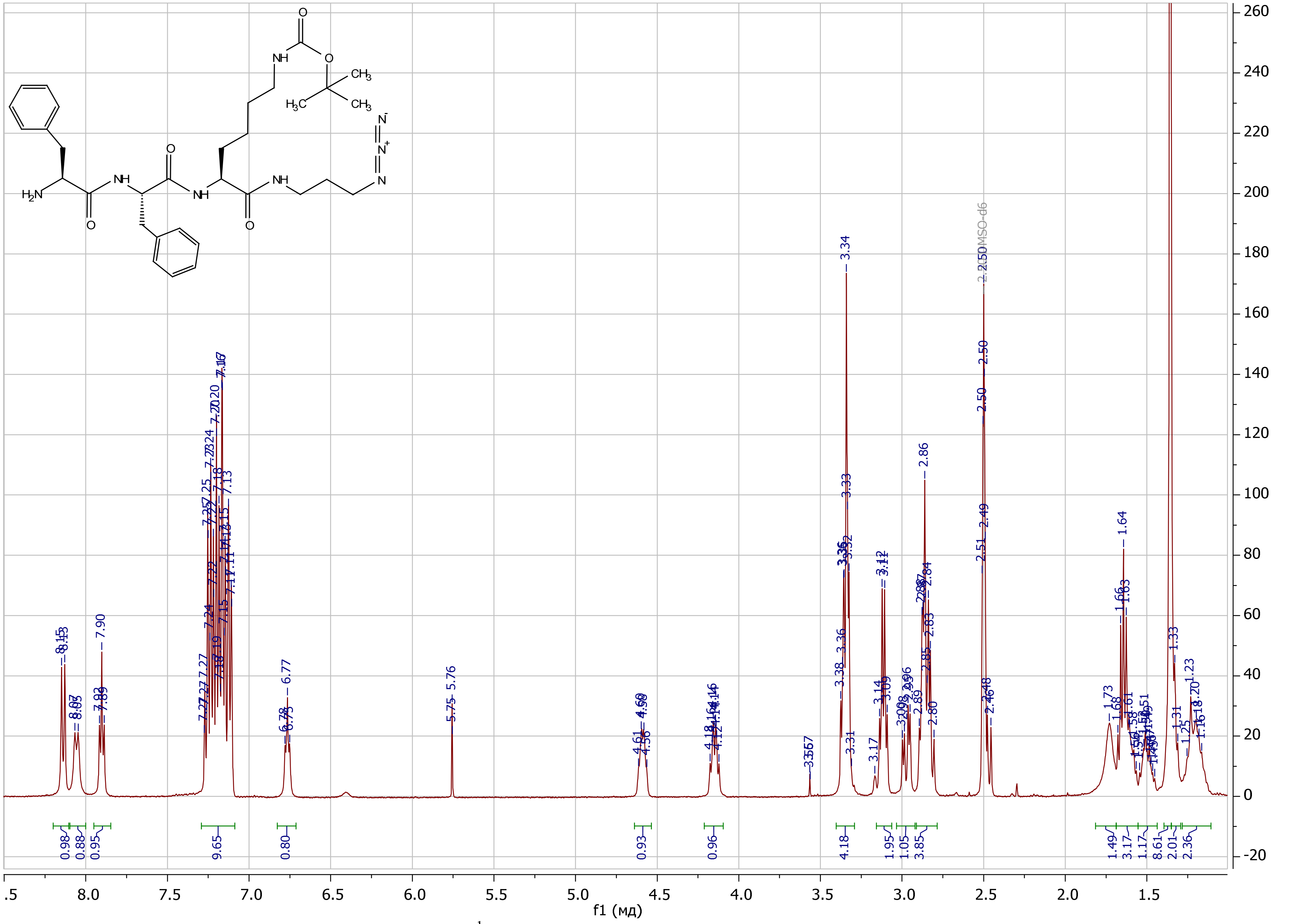

Figure S4. ^{1}H NMR spectrum of compound № **10** in DMSO-d_6

Compound 16.

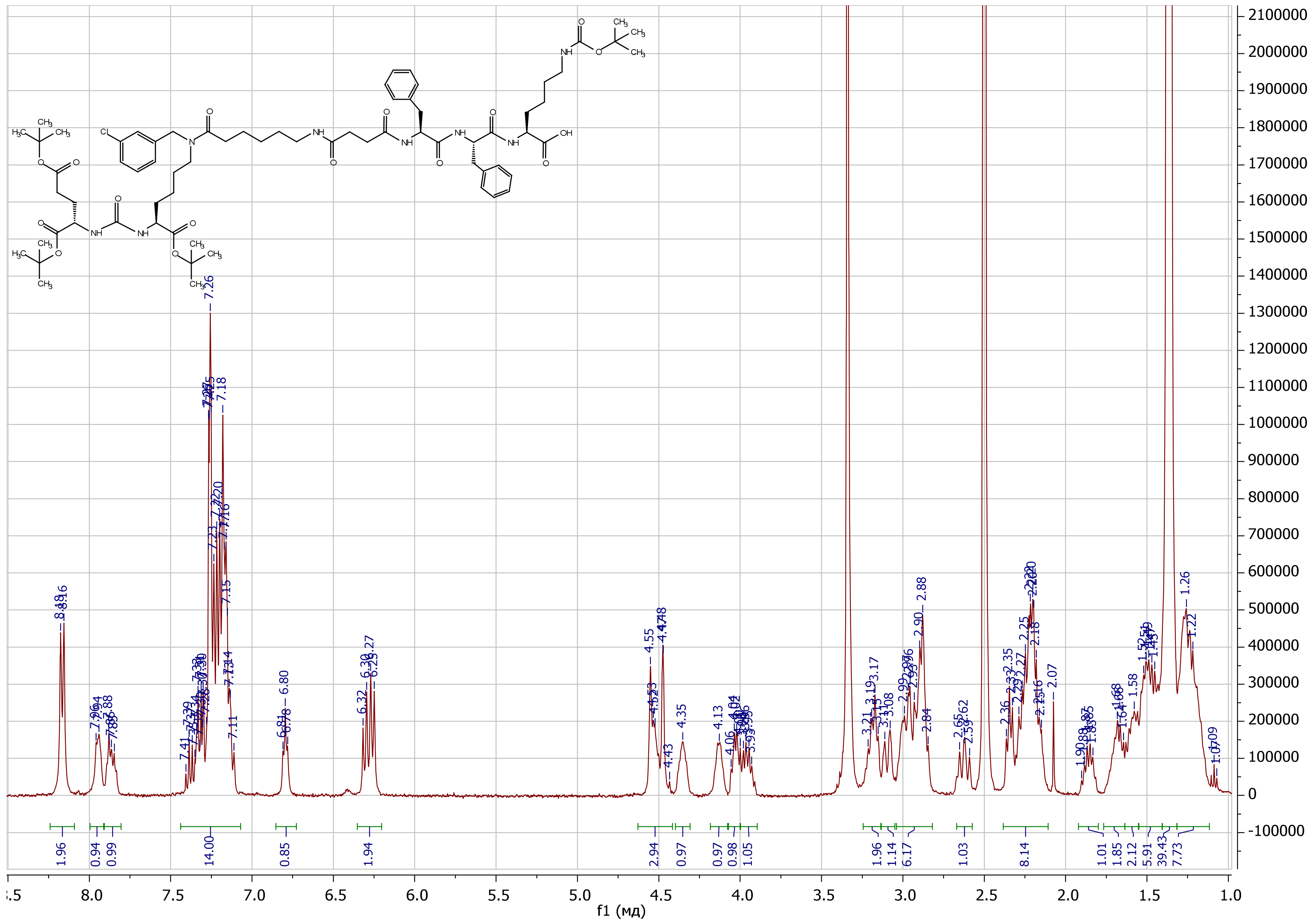

Figure S5. ^{1}H NMR spectrum of compound № **16** in DMSO-d_6

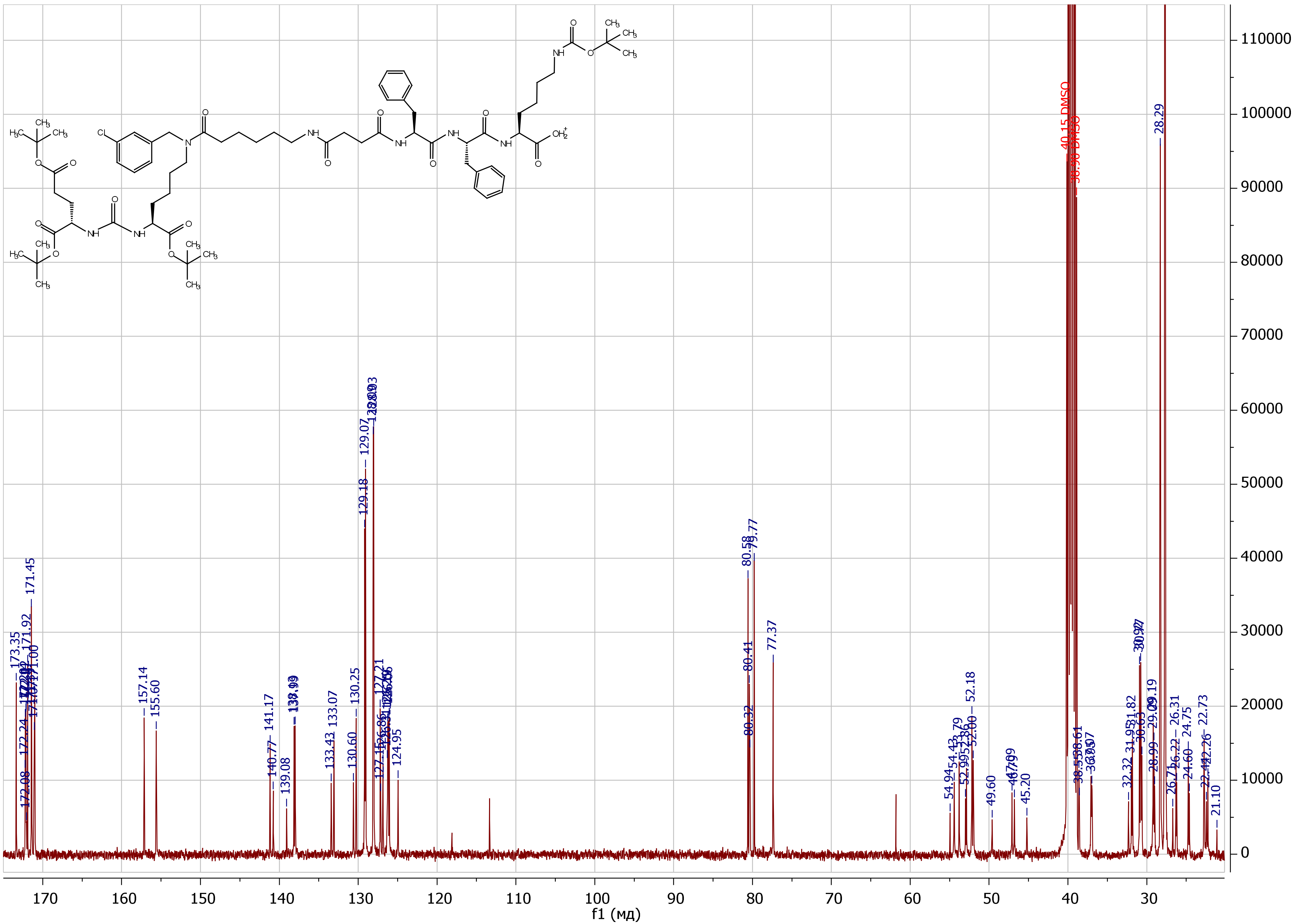

Figure S6. ^{13}C NMR spectrum of compound № **16** in DMSO-*d*$_6$

Compound 11.

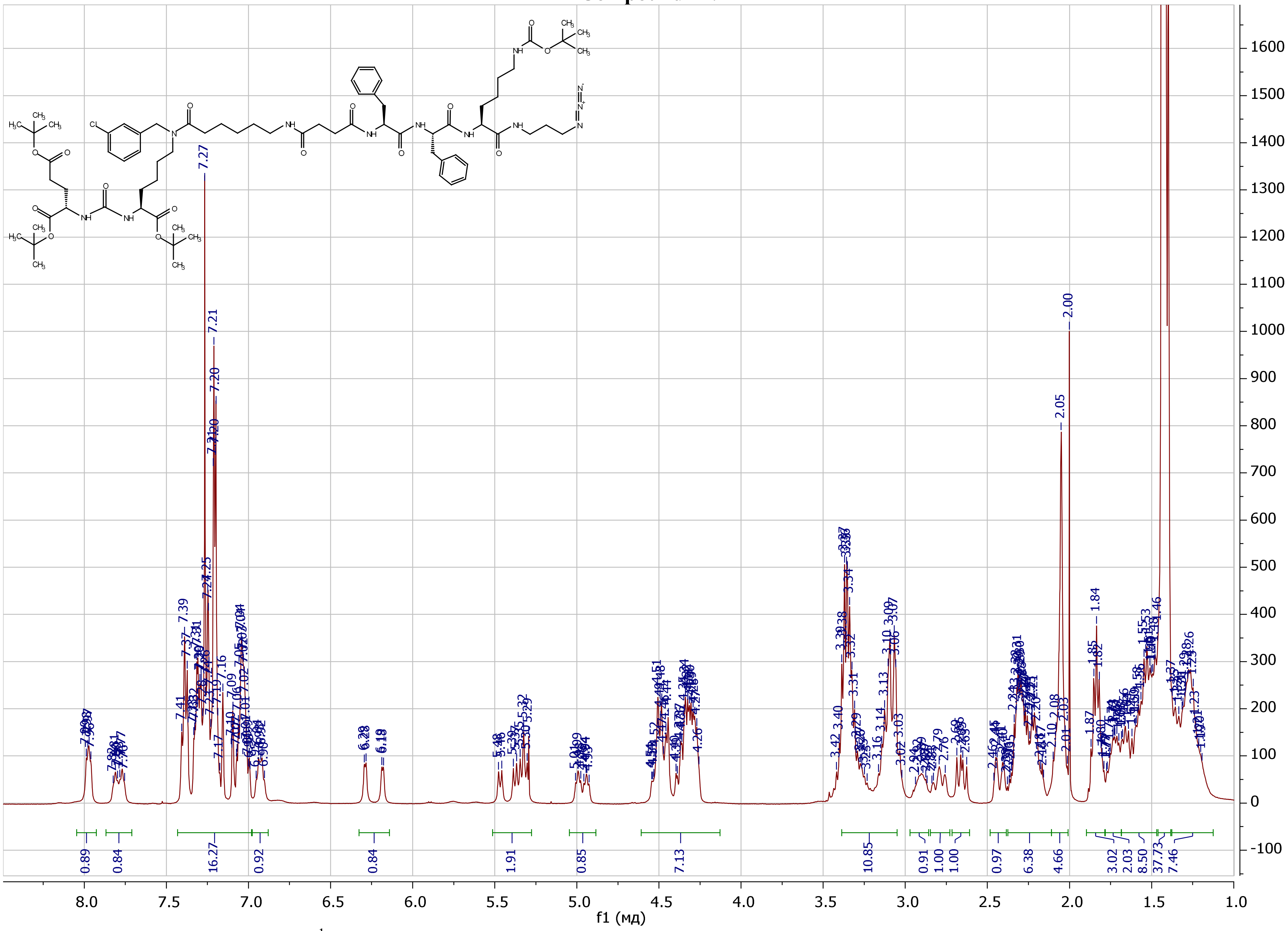

Figure S7. ^{1}H NMR spectrum of compound № **11** in $CDCl_3$. **Liquid-phase technique. Method 2.**

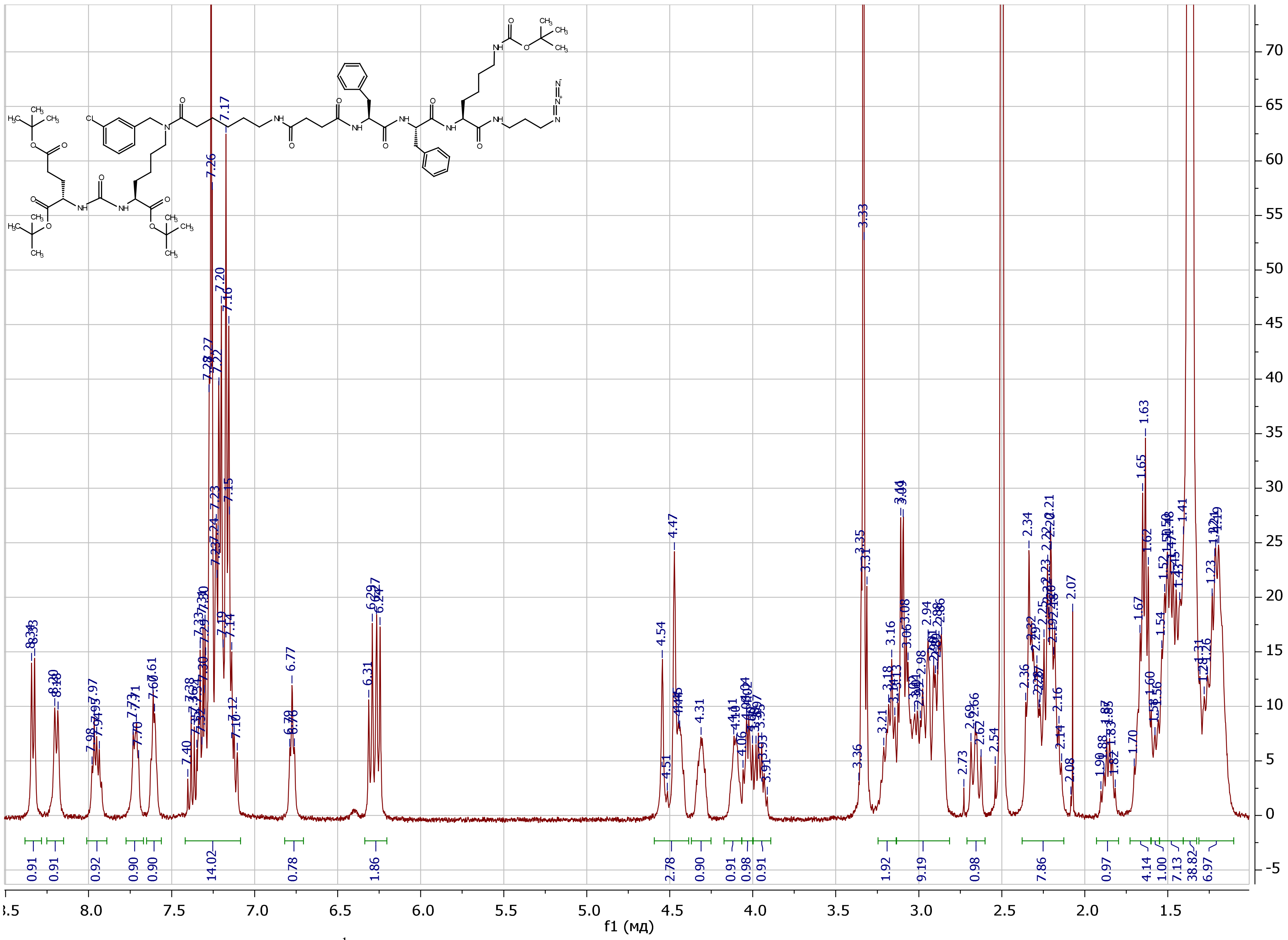

Figure S8. ^{1}H NMR spectrum of compound № **11** in DMSO-d_6. **SPPS technique. Method 1.**

Compound 12.

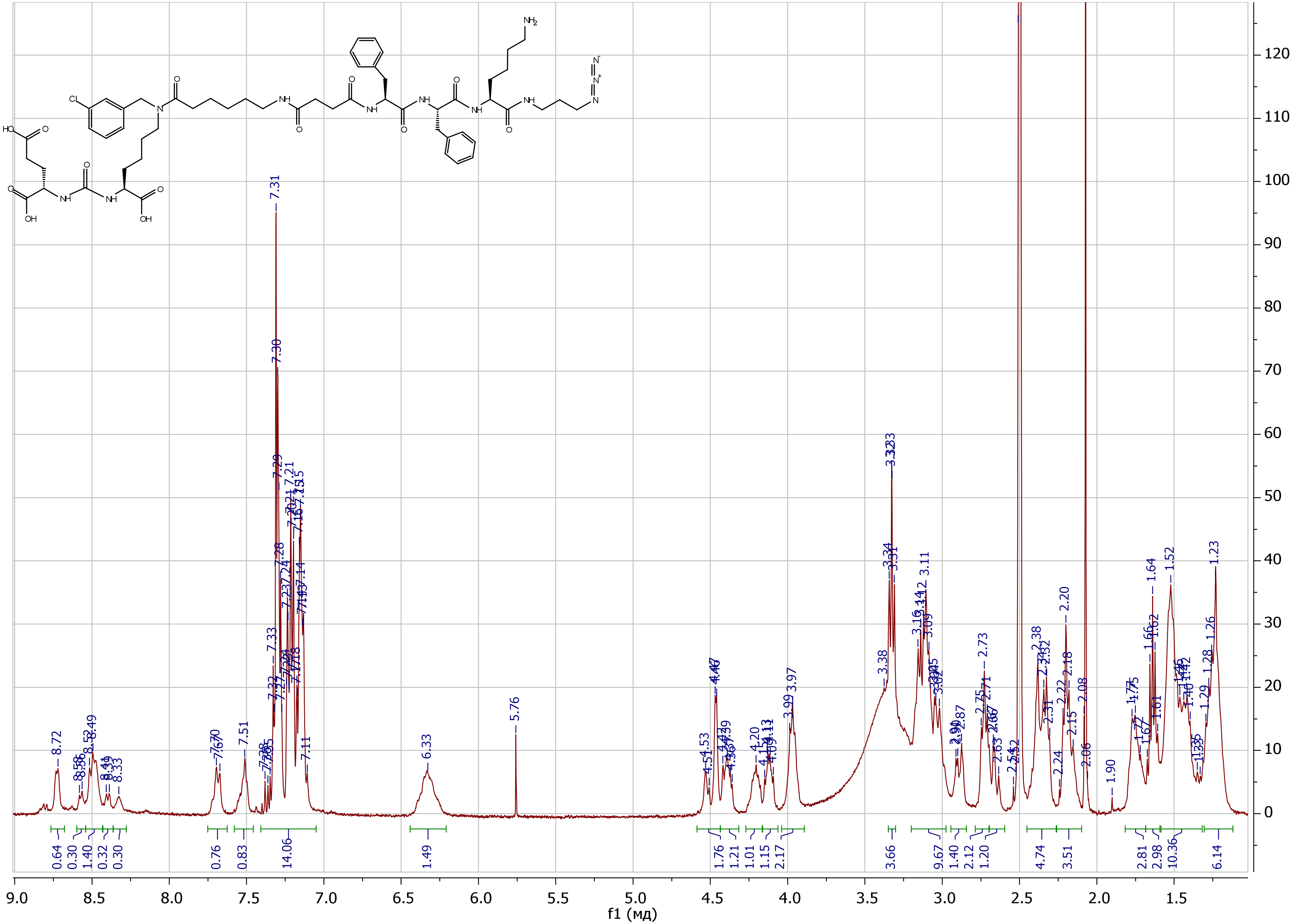

Figure S9. ^{1}H NMR spectrum of compound № **12** in DMSO-d_6. **Liquid-phase technique.**

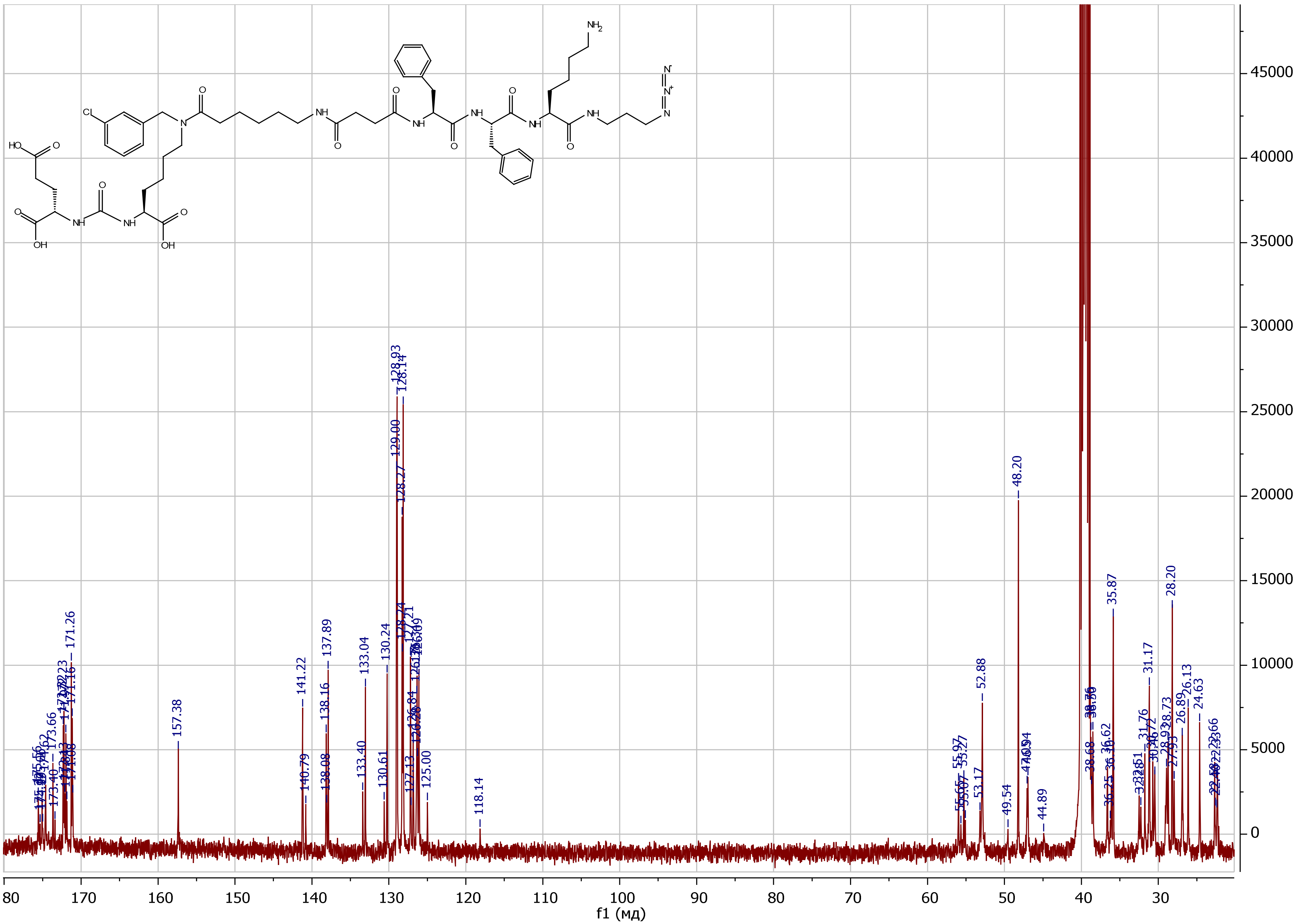

Figure S10. ^{13}C NMR spectrum of compound № **12** in DMSO-d_6. **Liquid-phase technique.**

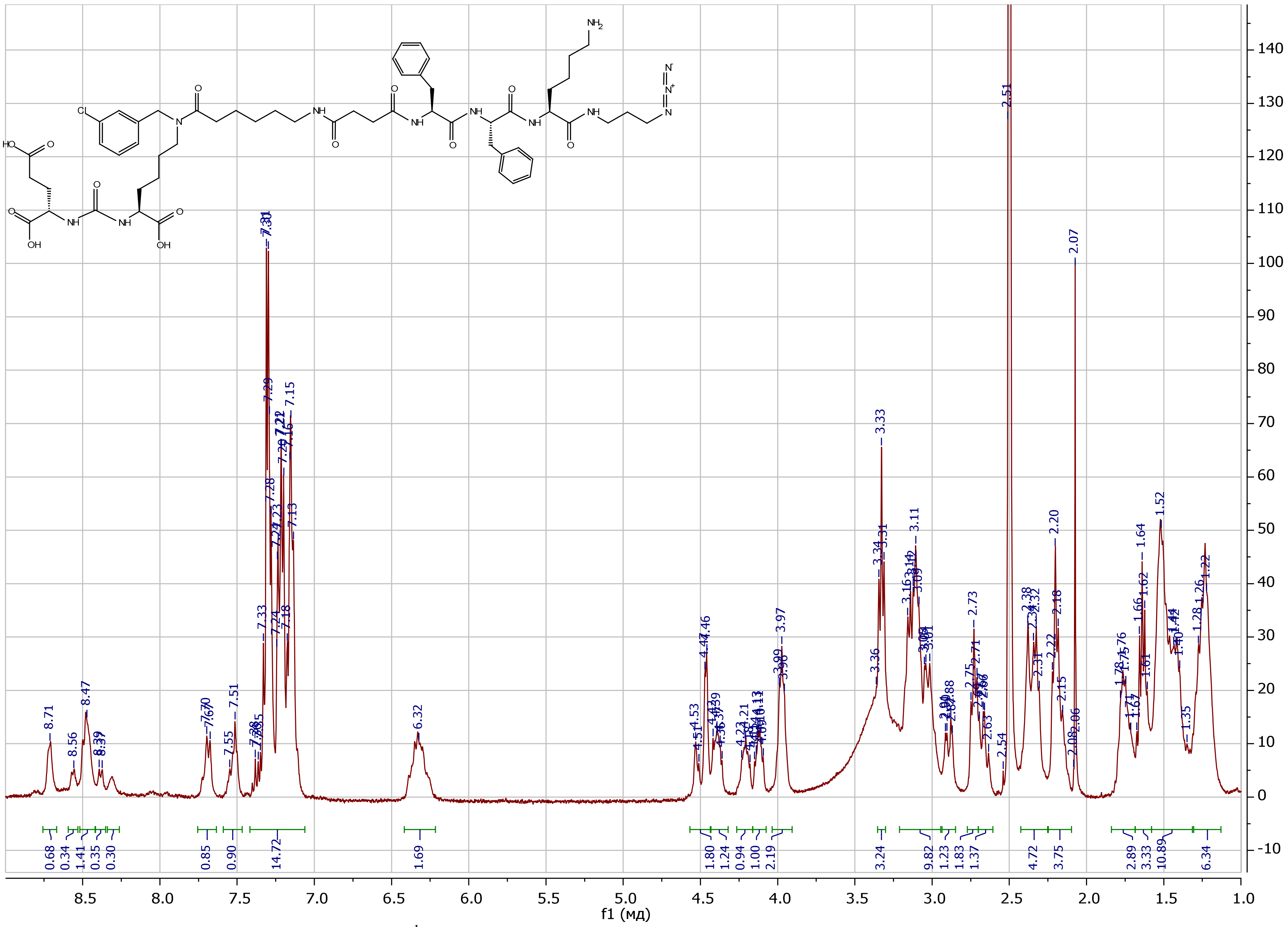

Figure S11. ^{1}H NMR spectrum of compound № **12** in DMSO-d_6. **SPPS technique.**

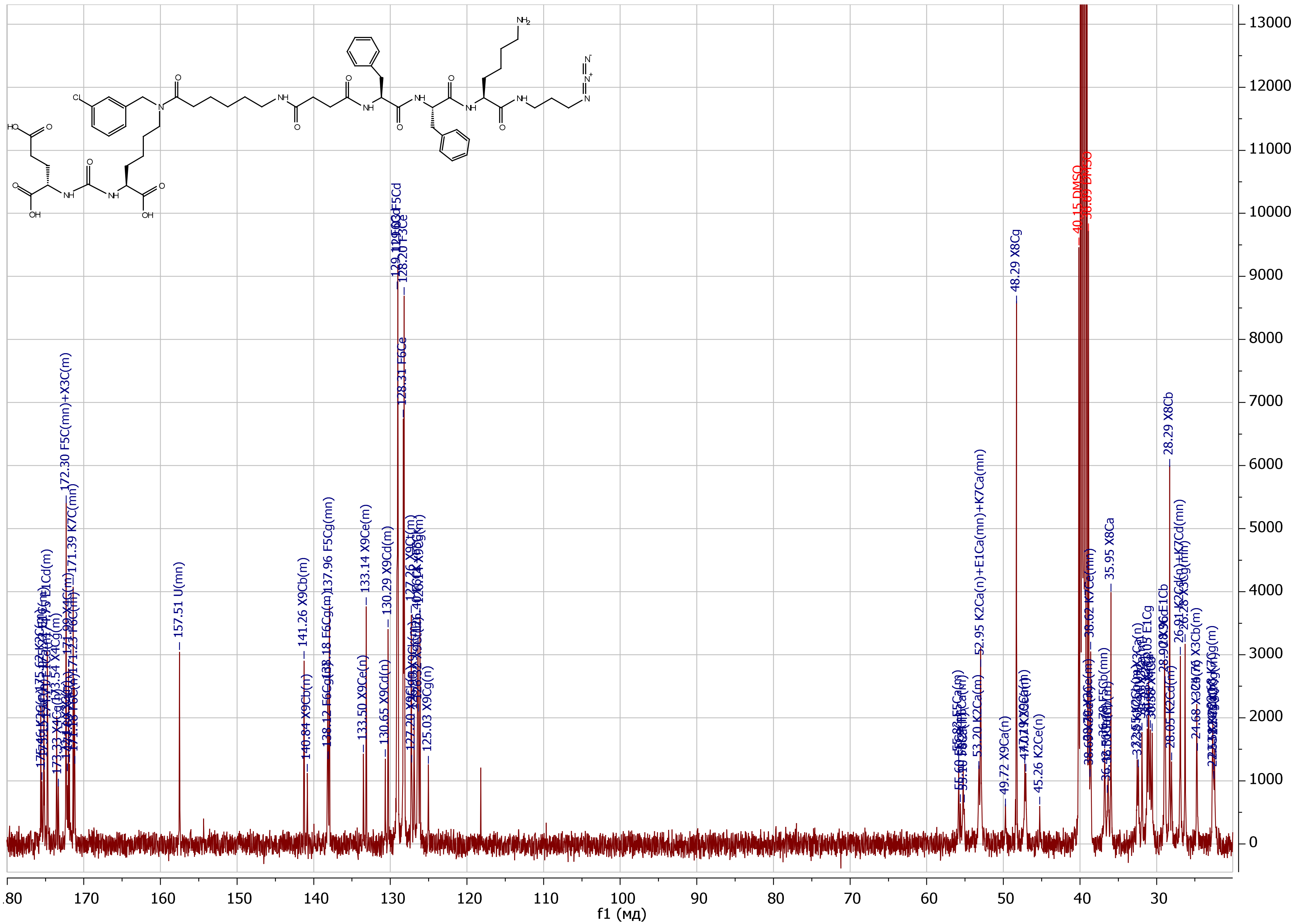

Figure S12. ^{13}C NMR spectrum of compound № **12** in DMSO-d_6. **SPPS technique.**

Compound 17.

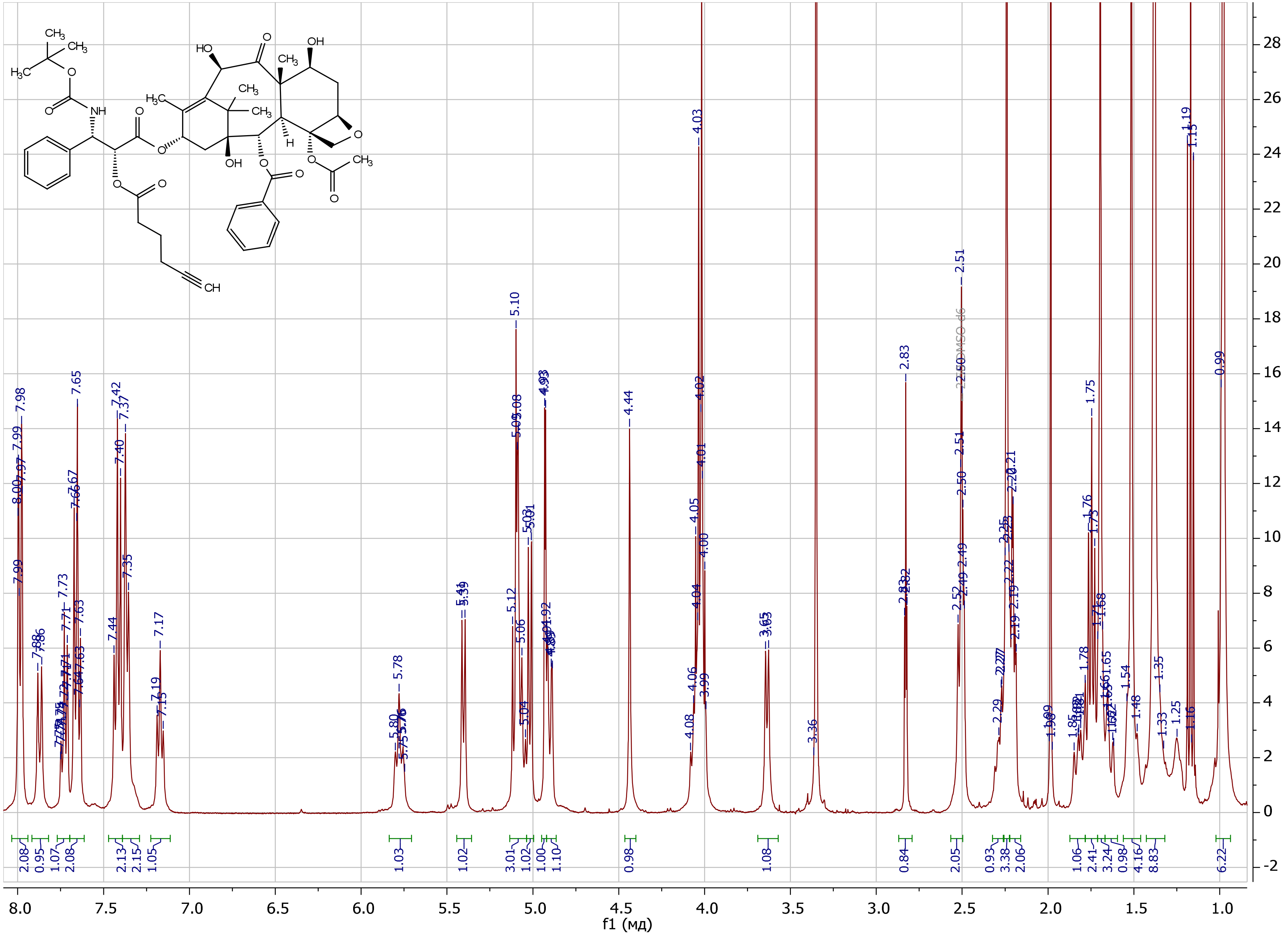

Figure S13. ^{1}H NMR spectrum of compound № **17** in DMSO-d_6

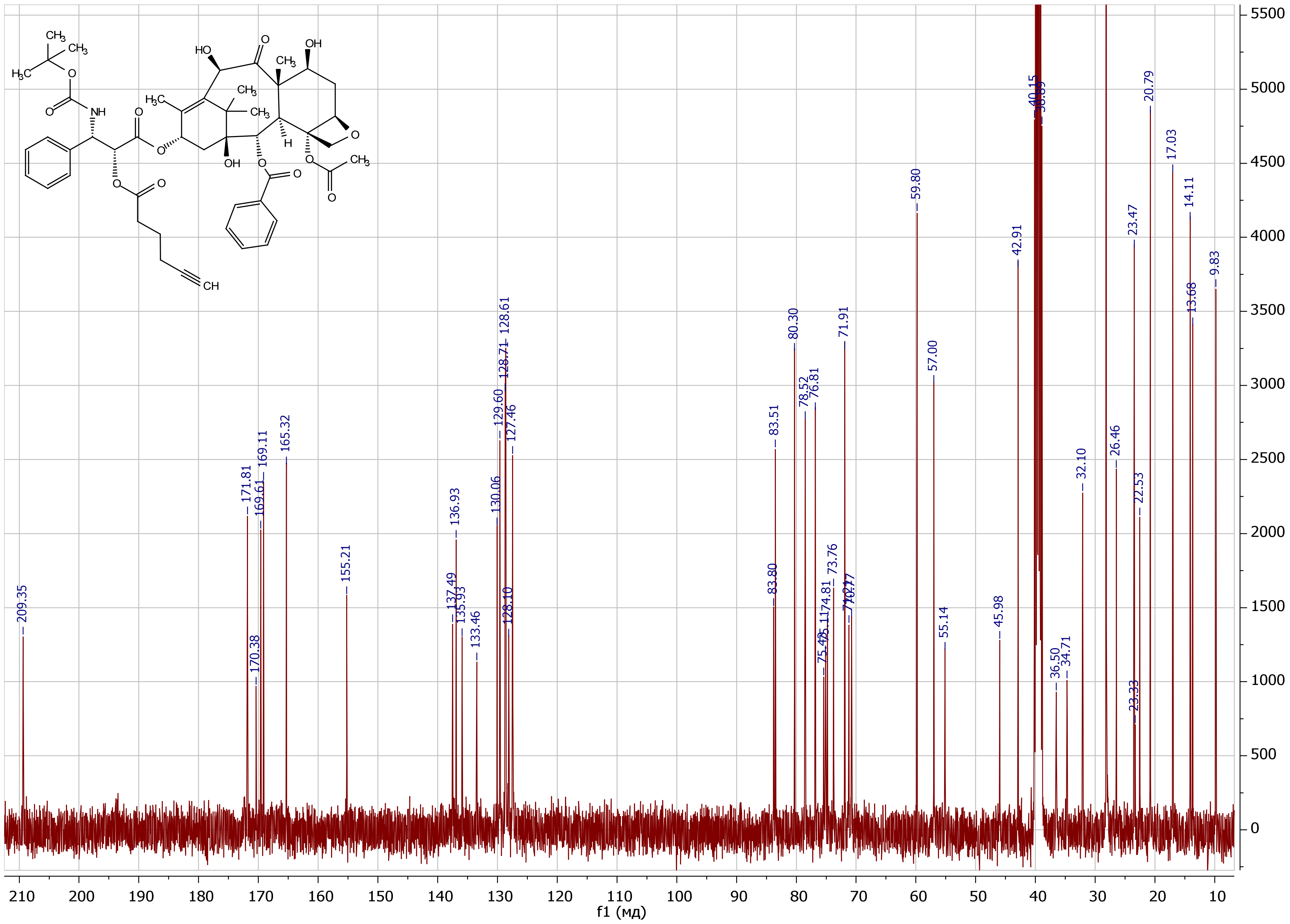

Figure S14. ^{13}C NMR spectrum of compound № **17** in DMSO-d_6

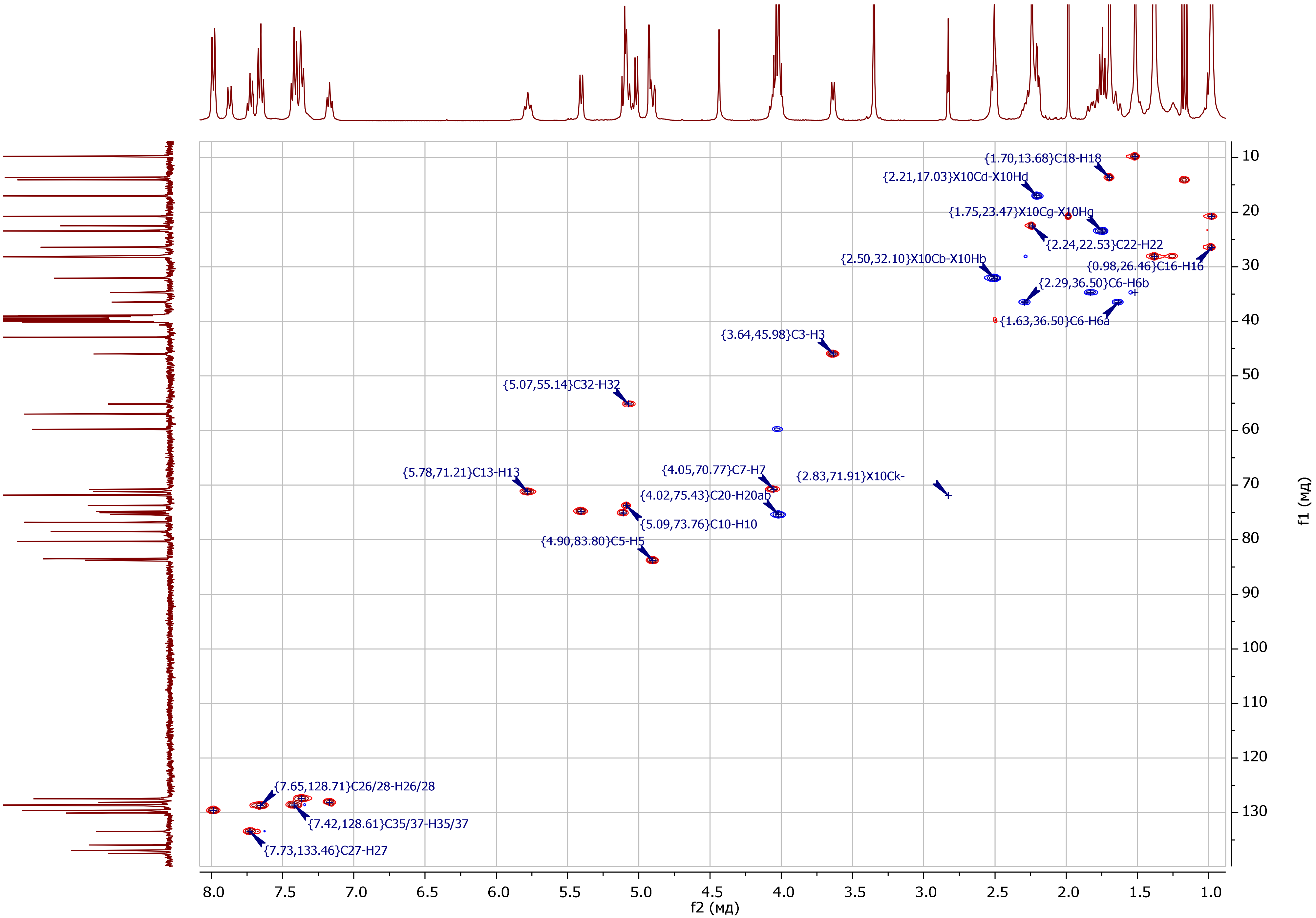

Figure S15. HSQC 1H-13C spectrum of compound № **17** in DMSO-d_6, T = 296° K.

Table S1. HSQC ($^{13}C\Delta\delta/^{1}H\Delta\delta$ (ppm/ppm)) of **17** in DMSO-d_6, T = 296° K.

	f1	f2	Int	Annotation
1	133.46	7.73	0.0	C27-H27
2	129.60	7.99	0.1	C25/ 29-H25/ 29
3	128.71	7.65	0.1	C26/ 28-H26/ 28
4	128.61	7.42	0.1	C35/ 37-H35/ 37
5	128.10	7.17	0.0	C36-H36
6	127.46	7.37	0.1	C34/ 38-H34/ 38
7	83.80	4.90	0.0	C5-H5
8	75.43	4.02	-0.1	C20-H20ab
9	75.11	5.11	0.0	C31-H31
10	74.81	5.40	0.1	C2-H2
11	73.76	5.09	0.1	C10-H10
12	71.91	2.83	-5.7	X10Ck-X10Hk
13	71.21	5.78	0.0	C13-H13
14	70.77	4.05	0.0	C7-H7
15	55.14	5.07	0.0	C32-H32
16	45.98	3.64	74.4	C3-H3
17	36.50	1.63	-24.1	C6-H6a
18	36.50	2.29	-27.9	C6-H6b
19	34.71	1.83	-30.2	C14-H14b
20	34.71	1.52	-4.1	C14-H14a
21	32.10	2.50	-88.8	X10Cb-X10Hb
22	28.16	1.38	89.2	tBu
23	26.46	0.98	63.6	C16-H16
24	23.47	1.75	-74.1	X10Cg-X10Hg
25	22.53	2.24	85.2	C22-H22
26	20.79	0.98	42.0	C17-H17
27	17.03	2.21	-76.2	X10Cd-X10Hd
28	13.68	1.70	93.1	C18-H18
29	9.83	1.52	86.2	C19-H19

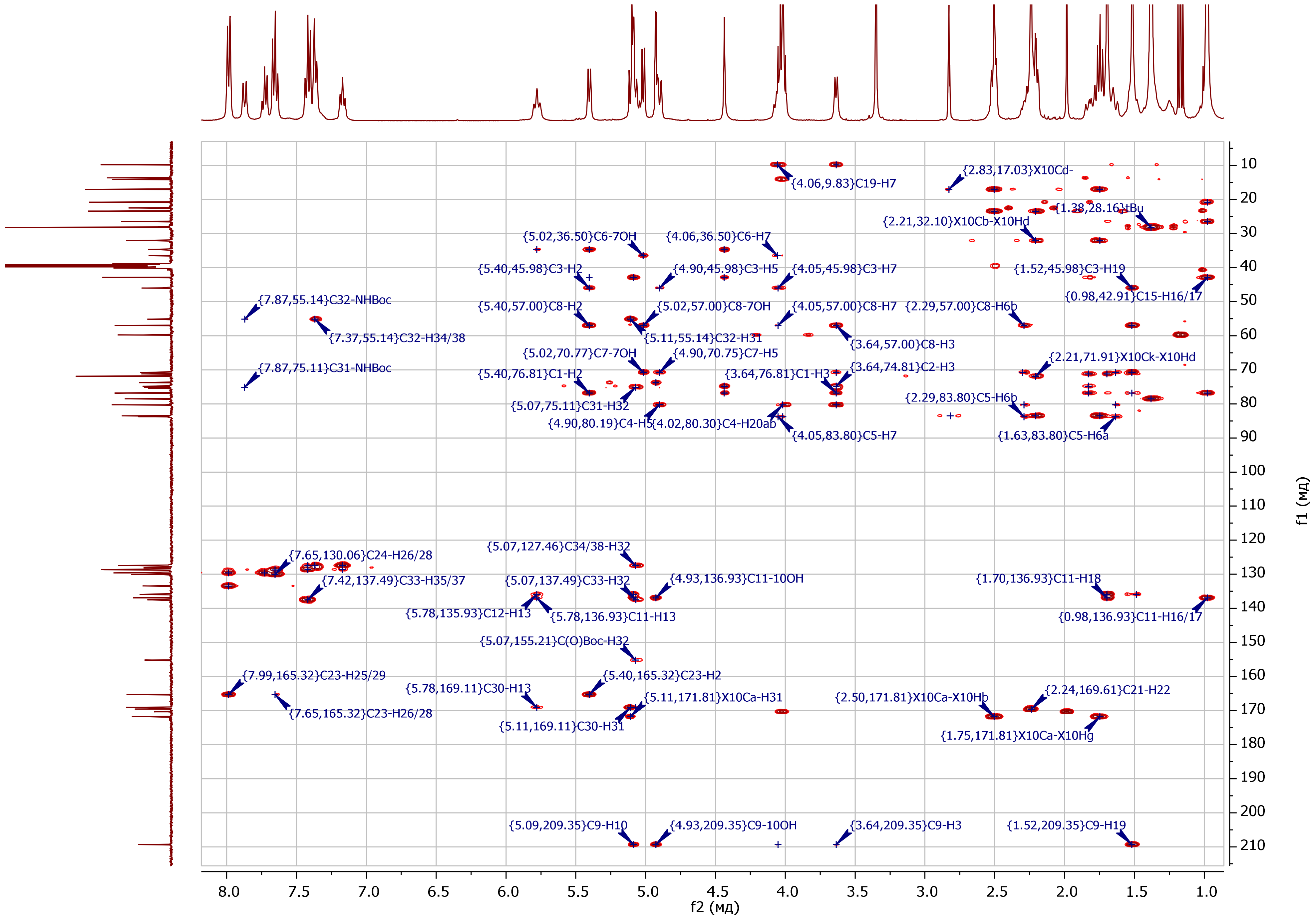

Figure S16. HMBC 1H-13C spectrum of compound № **17** in DMSO-d_6, T = 296° K.

Table S2. HMBC ($^{13}C\Delta\delta/^{1}H\Delta\delta$ (ppm/ppm)) of **17** in DMSO-d_6, T = 296° K.

	f1	f2	Int	Annotation
1	209.35	4.93	67.2	C9-10OH
2	209.35	3.64	6.3	C9-H3
3	209.35	4.05	3.9	C9-H7
4	209.35	5.09	44.3	C9-H10
5	209.35	1.52	95.6	C9-H19
6	171.81	5.11	31.4	X10Ca-H31
7	171.81	2.50	105.9	X10Ca-X10Hb
8	171.81	1.75	69.2	X10Ca-X10Hg
9	169.61	2.24	209.5	C21-H22
10	169.11	5.78	14.8	C30-H13
11	169.11	5.11	84.5	C30-H31
12	169.11	5.07	15.2	C30-H32
13	165.32	7.99	57.8	C23-H25/ 29
14	165.32	7.65	5.1	C23-H26/ 28
15	165.32	5.40	59.8	C23-H2
16	155.21	5.07	9.7	C(O)Boc-H32
17	137.49	7.42	111.6	C33-H35/ 37
18	137.49	5.07	25.6	C33-H32
19	136.93	5.09	54.3	C11-H10
20	136.93	5.78	16.1	C11-H13
21	136.93	4.93	72.3	C11-10OH
22	136.93	0.98	65.5	C11-H16/ 17
23	136.93	1.70	87.2	C11-H18
24	135.93	5.78	21.5	C12-H13
25	135.93	5.09	42.7	C12-H10
26	135.93	1.70	105.7	C12-H18
27	135.93	1.49	12.4	C12-H14a
28	133.46	7.99	73.6	C27-H25/ 29
29	130.06	7.65	115.3	C24-H26/ 28
30	129.60	7.99	53.2	C25/ 29-H25/ 29
31	129.60	7.73	114.0	C25/ 29-H27
32	128.71	7.65	70.8	C26/ 28-H26/ 28
33	128.61	7.42	80.5	C35/ 37-H35/ 37
34	128.61	7.17	20.8	C35/ 37-H36
35	127.46	7.37	71.2	C34/ 38-H34/ 38
36	127.46	5.07	23.9	C34/ 38-H32
37	127.46	7.17	123.1	C34/ 38-H36
38	127.46	7.42	18.6	C34/ 38-H35/ 37
39	83.80	4.02	10.9	C5-H20ab
40	83.80	4.05	9.7	C5-H7
41	83.80	2.29	6.9	C5-H6b
42	83.80	1.63	12.6	C5-H6a
43	83.51	2.21	70.9	X10Ce-X10Hd
44	83.51	2.82	1.1	X10Ce-X10Hk
45	83.51	1.75	89.2	X10Ce-X10Hg
46	80.30	3.64	49.5	C4-H3
47	80.30	4.02	35.7	C4-H20ab
48	80.30	1.63	7.8	C4-H6a
49	80.29	2.29	1.7	C4-H6b
50	80.19	4.90	8.1	C4-H5
51	78.52	1.38	117.7	tBu-CBoc
52	76.81	4.44	32.5	C1-1OH
53	76.81	5.40	58.5	C1-H2
54	76.81	3.64	34.8	C1-H3
55	76.81	1.52	5.7	C1-H14a
56	76.81	1.83	17.5	C1-H14b
57	76.81	0.98	73.0	C1-H16/ 17
58	75.11	7.87	2.3	C31-NHBoc
59	75.11	5.07	17.3	C31-H32
60	74.81	4.44	64.1	C2-1OH
61	74.81	3.64	39.8	C2-H3
62	74.81	1.83	13.9	C2-H14b
63	73.76	4.93	37.6	C10-10OH
64	71.91	2.21	43.3	X10Ck-X10Hd
65	71.21	1.83	24.1	C13-H14b
66	71.21	1.70	38.2	C13-H18
67	70.77	5.02	53.1	C7-7OH
68	70.77	1.63	11.4	C7-H6a
69	70.75	3.64	12.5	C7-H3
70	70.75	1.52	61.2	C7-H19
71	70.75	2.30	8.8	C7-H6b
72	70.75	4.90	7.6	C7-H5
73	57.00	5.02	54.8	C8-7OH
74	57.00	5.40	63.7	C8-H2
75	57.00	4.05	3.8	C8-H7
76	57.00	2.29	21.3	C8-H6b
77	57.00	1.52	99.2	C8-H19
78	57.00	3.64	52.1	C8-H3
79	55.14	7.37	43.6	C32-H34/ 38
80	55.14	7.87	2.2	C32-NHBoc
81	55.14	5.11	109.2	C32-H31
82	45.98	4.90	3.6	C3-H5
83	45.98	1.52	46.5	C3-H19
84	45.98	4.05	9.1	C3-H7
85	45.98	5.40	24.2	C3-H2
86	42.91	5.09	59.1	C15-H10
87	42.91	5.40	4.7	C15-H2
88	42.91	4.44	20.4	C15-1OH
89	42.91	0.98	70.5	C15-H16/ 17
90	36.50	5.02	24.2	C6-7OH
91	36.50	4.06	5.3	C6-H7
92	34.71	5.40	32.0	C14-H2
93	34.71	5.78	7.7	C14-H13
94	34.71	4.44	54.4	C1-1OH
95	32.10	1.75	57.1	X10Cb-X10Hg
96	32.10	2.21	42.0	X10Cb-X10Hd
97	28.16	1.38	174.6	tBu
98	26.46	0.98	38.2	C16-H17
99	23.47	2.21	57.2	X10Cg-X10Hd
100	23.47	2.50	71.9	X10Cg-X10Hb
101	20.79	0.98	33.1	C17-H16
102	17.03	2.50	62.0	X10Cd-X10Hb
103	17.03	1.75	54.8	X10Cd-X10Hg
104	17.03	2.83	7.9	X10Cd-X10Hk
105	9.83	3.64	42.9	C19-H3
106	9.83	4.06	30.6	C19-H7

Compound 18.

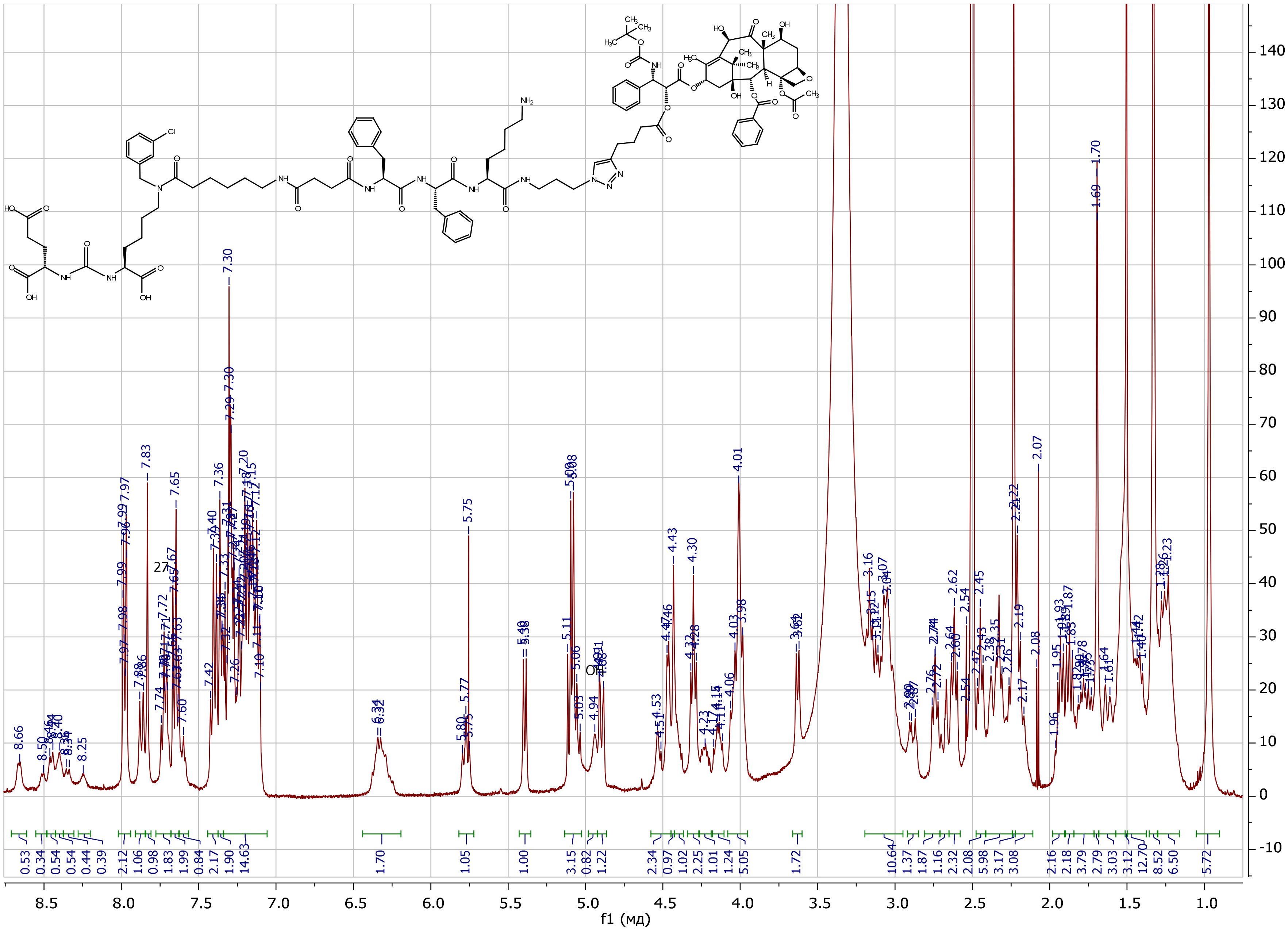

Figure S17. ^{1}H NMR spectrum of compound № **18** in DMSO-d_6

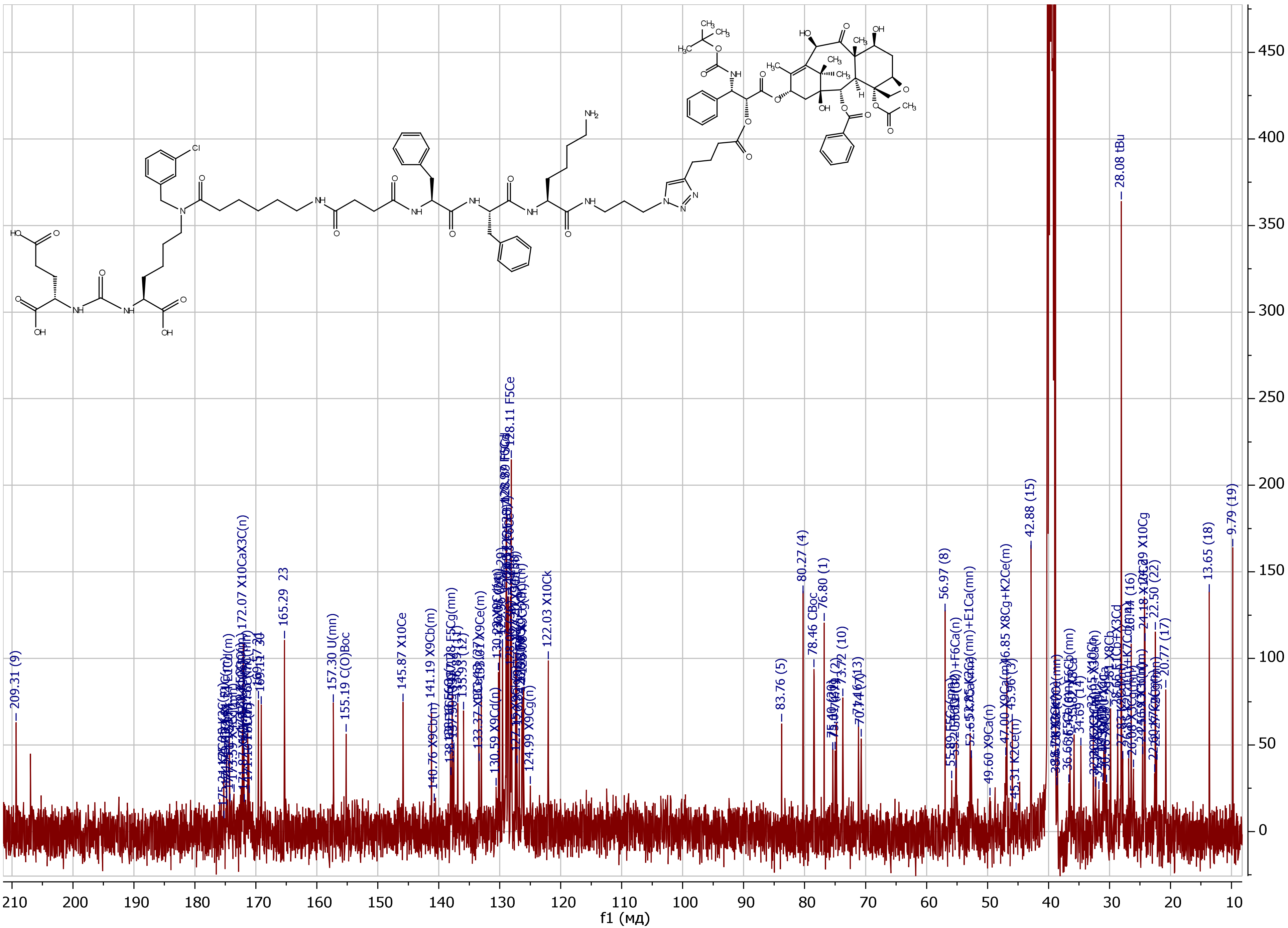

Figure S18. ^{13}C NMR spectrum of compound № **18** in DMSO-d_6

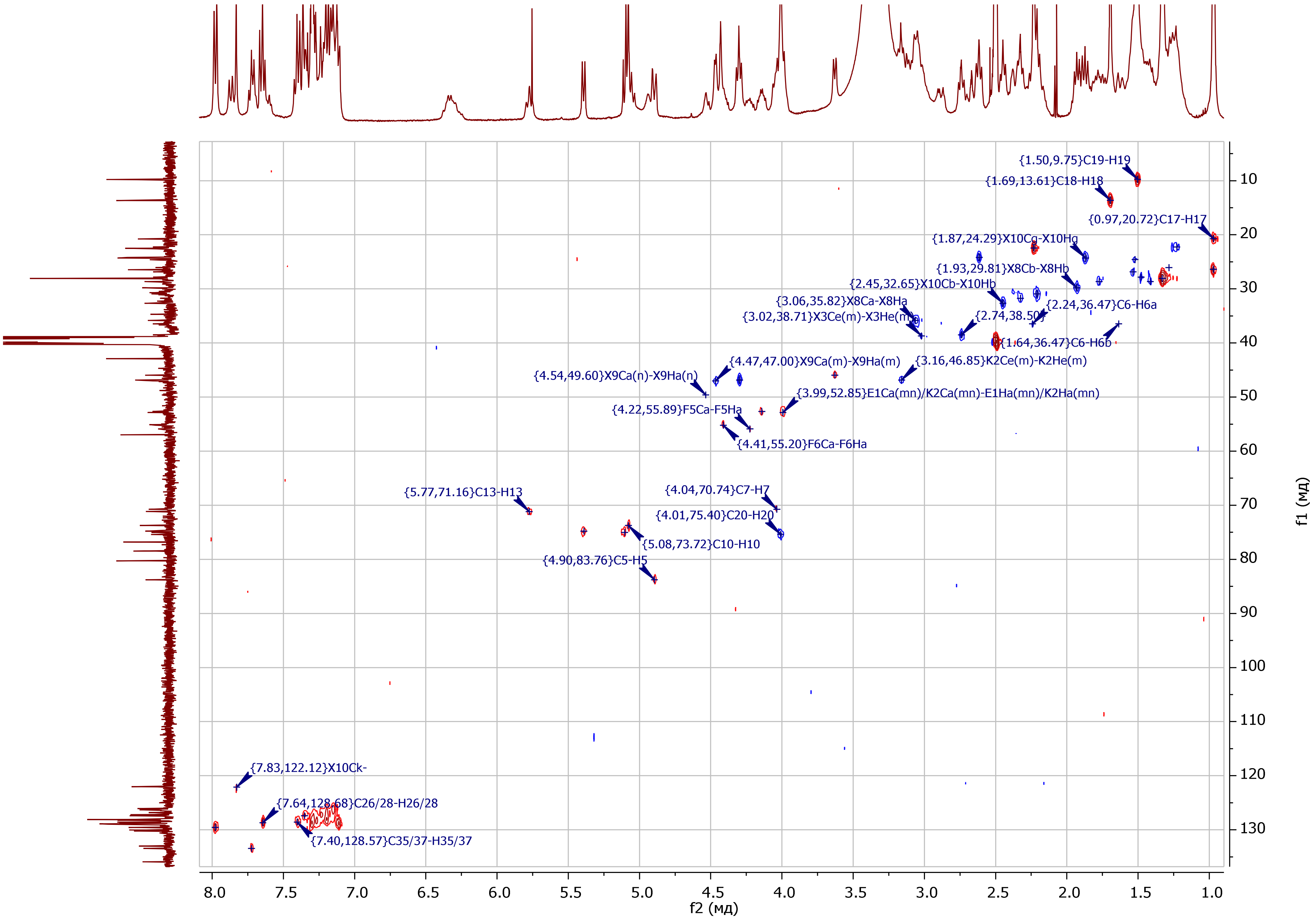

Figure S19. HSQC 1H-13C spectrum of compound № **18** in DMSO-d_6, T = 296° K.

Table S3. HSQC (^{13}CΔδ/^{1}HΔδ (ppm/ppm)) of **18** in DMSO-d_6, T = 296º K.

	f1	f2	Int	Annotation
1	133.43	7.72	5.8	C27-H27
2	129.56	7.98	12.4	C25/ 29-H25/ 29
3	128.68	7.64	8.4	C26/ 28-H26/ 28
4	128.57	7.40	9.8	C35/ 37-H35/ 37
5	127.41	7.35	11.7	C34/ 38-H34/ 38
6	122.12	7.83	1.6	X10Ck-X10Hk
7	83.76	4.90	5.4	C5-H5
8	75.40	4.01	-8.2	C20-H20
9	75.01	5.10	6.2	C31-H31
10	74.79	5.39	5.3	C2-H2
11	73.72	5.08	8.0	C10-H10
12	71.16	5.77	5.5	C13-H13
13	70.74	4.04	3.2	C7-H7
14	55.89	4.22	5.2	F5Ca-F5Ha
15	55.20	4.41	5.8	F6Ca-F6Ha
16	52.85	3.99	5.8	E1Ca(mn)/ K2Ca(mn)-E1Ha(mn)/ K2Ha(mn)
17	52.65	4.14	5.8	K7Ca(mn)-K7Ha
18	49.60	4.54	-2.7	X9Ca(n)-X9Ha(n)
19	47.00	4.47	-5.8	X9Ca(m)-X9Ha(m)
20	46.85	4.30	-16.6	X8Cg-X8Hg
21	46.85	3.16	-6.6	K2Ce(m)-K2He(m)
22	45.96	3.63	5.8	C3-H3
23	38.71	3.02	-5.0	X3Ce(m)-X3He(m)
24	38.50	2.74	-9.2	K7Ce-K7He
25	36.47	1.64	-1.2	C6-H6b
26	36.47	2.24	-1.2	C6-H6a
27	35.82	3.06	-10.4	X8Ca-X8Ha
28	32.65	2.45	-12.5	X10Cb-X10Hb
29	31.74	2.33	-8.7	X3Ca(m)-X3Ha(m)
30	30.92	2.21	-13.2	E1Cg-E1Hg
31	29.81	1.93	-12.7	X8Cb-X8Hb
32	28.66	1.77	-6.2	E1Cb-E1Hb(ab)
33	28.66	1.41	-6.2	X3Cd-X3Hd
34	28.08	1.33	50.1	tBu-tBu
35	27.83	1.48	-5.6	K2Cd(m)-K2Hd(m)
36	26.85	1.54	-5.8	K7Cd-K7Hd
37	26.40	0.97	18.4	C16-H16
38	26.08	1.28	-3.7	X3Cg(mn)-X3Hg(mn)
39	24.59	1.52	-4.4	X3Cb(m)-X3Hb(m)
40	24.29	1.87	-14.1	X10Cg-X10Hg
41	24.18	2.61	-11.1	X10Cd-X10Hd
42	22.45	2.23	21.6	C22-H22
43	22.27	1.23	-8.0	K2Cg-K2Hg
44	20.72	0.97	12.1	C17-H17
45	13.61	1.69	24.6	C18-H18
46	9.75	1.50	27.1	C19-H19

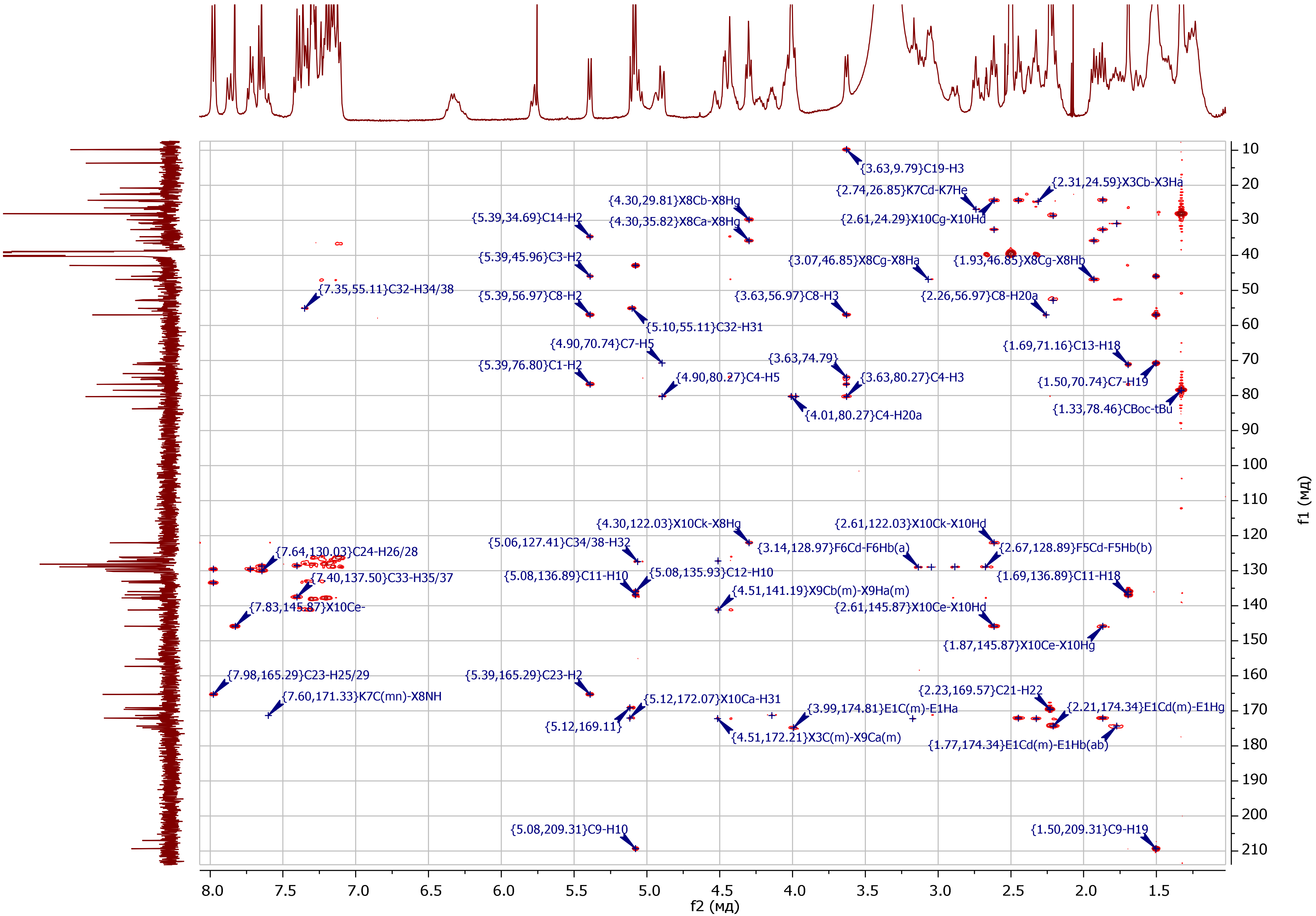

Figure S20. HMBC 1H-13C spectrum of compound № **18** in DMSO-d_6, T = 296º K.

Table S4. HMBC ($^{13}C\Delta\delta/^{1}H\Delta\delta$ (ppm/ppm)) of **18** in DMSO-d_6, T = 296º K.

	f1	f2	Int	Annotation
1	209.31	1.50	1.9	C9-H19
2	209.31	5.08	0.5	C9-H10
3	174.81	3.99	0.2	E1C(m)-E1Ha
4	174.34	1.77	0.1	E1Cd(m)-E1Hb(ab)
5	174.34	2.21	0.6	E1Cd(m)-E1Hg
6	172.21	4.51	0.1	X3C(m)-X9Ca(m)
7	172.21	2.33	0.2	X3C-X3Ha(m)
8	172.21	3.17	0.1	X3C-K2Ce(m)
9	172.07	5.12	0.2	X10Ca-H31
10	172.07	2.45	0.4	X10Ca-X10Hb
11	172.07	1.87	0.3	X10Ca-X10Hg
12	171.33	4.14	0.1	K7C-K7Ha
13	171.33	7.60	0.1	K7C(mn)-X8NH
14	169.57	2.23	2.2	C21-H22
15	169.11	5.12	0.4	C30-H31
16	165.29	7.98	0.1	C23-H25/ 29
17	165.29	5.39	0.3	C23-H2
18	145.87	2.61	0.4	X10Ce-X10Hd
19	145.87	7.83	0.3	X10Ce-X10Hk
20	145.87	1.87	0.2	X10Ce-X10Hg
21	141.19	4.51	0.1	X9Cb(m)-X9Ha(m)
22	137.50	7.40	0.3	C33-H35/ 37
23	136.89	5.08	0.6	C11-H10
24	136.89	1.69	1.4	C11-H18
25	136.89	0.97	2.2	C11-H16/ 17
26	135.93	5.08	0.5	C12-H10
27	135.93	1.69	1.5	C12-H18
28	133.43	7.98	0.4	C27-H25/ 29
29	130.03	7.64	0.4	C24-H26/ 28
30	129.56	7.98	0.2	C25/ 29-H25/ 29
31	129.56	7.72	0.3	C27-H25/ 29
32	128.97	3.14	0.1	F6Cd-F6Hb(a)
33	128.97	3.05	0.0	F6Cd-F6Hd(b)
34	128.89	2.89	0.0	F5Cd-F5Hb(a)
35	128.89	2.67	0.2	F5Cd-F5Hb(b)
36	128.68	7.64	0.3	C26/ 28-H26/ 28
37	128.57	7.40	0.3	C35/ 37-H35/ 37
38	127.41	5.06	0.1	C34/ 38-H32
39	127.20	4.51	0.1	X9Ct(m)-X9Ca(m)
40	122.03	4.30	0.2	X10Ck-X8Hg
41	122.03	2.61	0.2	X10Ck-X10Hd
42	80.27	4.90	0.1	C4-H5
43	80.27	3.98	0.1	C4-H20b
44	80.27	3.63	0.3	C4-H3
45	80.27	4.01	0.2	C4-H20a
46	78.46	1.33	5.6	CBoc-tBu
47	76.80	5.39	0.3	C1-H2
48	76.80	3.63	0.2	C1-H3
49	76.80	0.97	2.0	C1-H16/ 17
50	74.79	3.63	0.2	C2-H3
51	71.16	1.69	0.6	C13-H18
52	70.74	4.90	0.0	C7-H5
53	70.74	1.50	1.2	C7-H19
54	56.97	3.63	0.3	C8-H3
55	56.97	2.26	0.1	C8-H20a
56	56.97	1.50	2.1	C8-H19
57	56.97	5.39	0.3	C8-H2
58	55.11	7.35	0.1	C32-H34/ 38
59	55.11	5.10	0.1	C32-H31
60	52.85	2.21	0.1	E1Ca-E1Hg
61	46.85	3.07	0.1	X8Cg-X8Ha
62	46.85	1.93	0.3	X8Cg-X8Hb
63	45.96	5.39	0.1	C3-H2
64	45.96	1.50	1.1	C3-H19
65	42.88	5.08	0.6	C15-H10
66	42.88	0.97	2.6	C15-H16/ 17
67	35.82	4.30	0.3	X8Ca-X8Hg
68	35.82	1.93	0.2	X8Ca-X8Hb
69	34.69	5.39	0.2	C14-H2
70	32.65	2.61	0.2	X10Cb-X10Hd
71	32.65	1.87	0.2	X10Cb-X10Hg
72	30.92	1.77	0.1	E1Cg-E1Hb(b)
73	29.81	4.30	0.3	X8Cb-X8Hg
74	28.66	2.21	0.2	E1Cb-E1Hg
75	26.85	2.74	0.1	K7Cd-K7He
76	26.44	0.97	0.8	C16-H17
77	24.59	2.31	0.1	X3Cb-X3Ha
78	24.29	2.61	0.2	X10Cg-X10Hd
79	24.29	2.45	0.4	X10Cg-X10Hb
80	24.18	1.87	0.2	X10Cd-X10Hg
81	20.77	0.97	1.0	C17-H16
82	9.79	3.63	0.3	C19-H3

Compound 19.

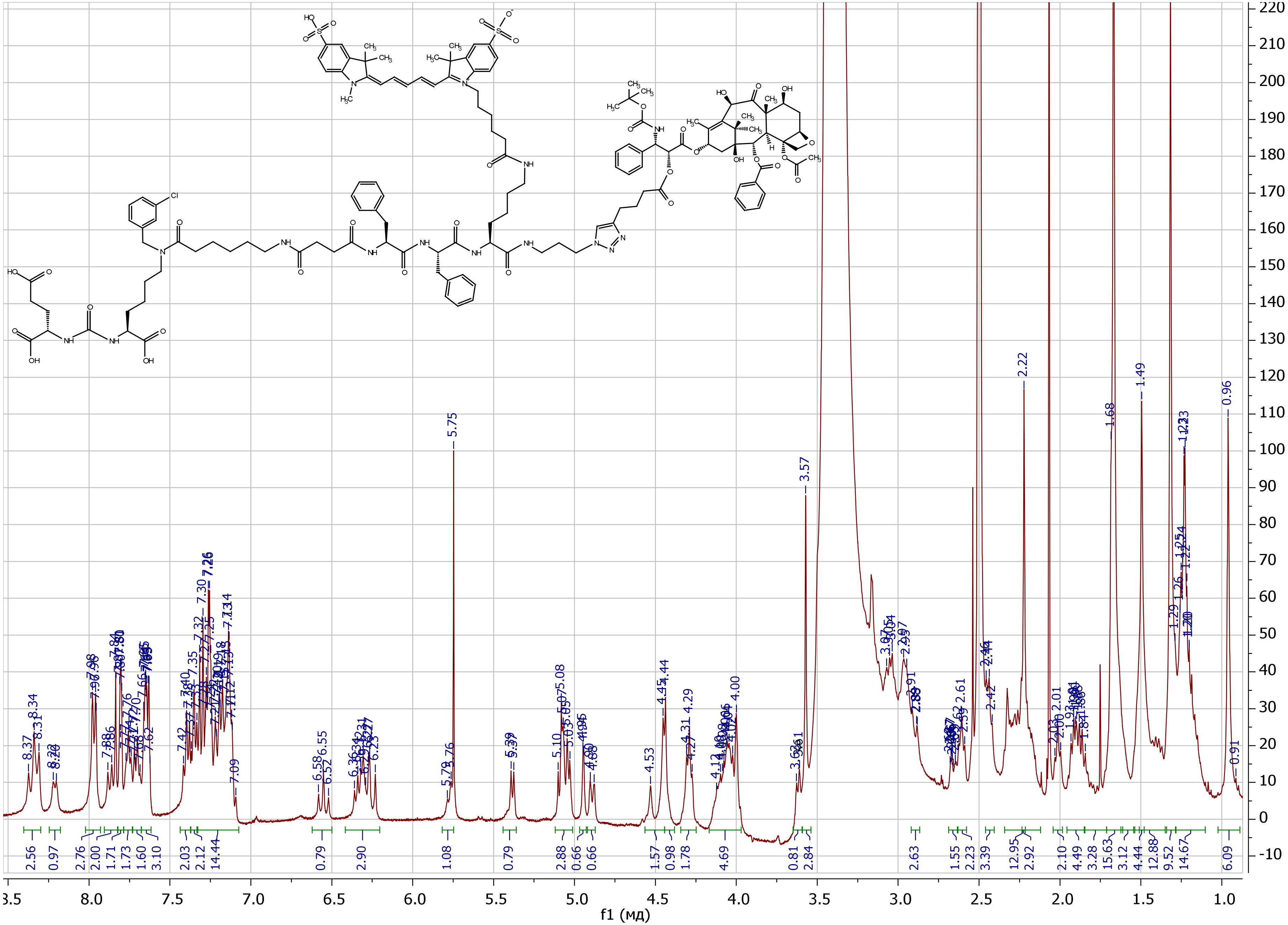

Figure S21. ^{1}H NMR spectrum of compound № **19** in DMSO-d_6
